# Functional phenotypes in schizophrenia spectrum disorders: defining the constructs and identifying biopsychosocial correlates using data-driven methods

**DOI:** 10.1038/s41537-024-00479-9

**Published:** 2024-06-24

**Authors:** Sunny X. Tang, Katrin Hänsel, Lindsay D. Oliver, Erin W. Dickie, Colin Hawco, Majnu John, Aristotle Voineskos, James M. Gold, Robert W. Buchanan, Anil K. Malhotra

**Affiliations:** 1 Division of Psychiatry Research, Feinstein Institutes for Medical Research, Northwell Health, New Hyde Park, NY USA; 2 Department of Psychiatry, Zucker Hillside Hospital, Northwell Health, New Hyde Park, NY USA; 3 Donald and Barbara Zucker School of Medicine at Hofstra/Northwell, Uniondale, NY USA; 4 Campbell Family Mental Health Research Institute, The Centre for Addiction and Mental Health, Toronto, ON Canada; 5 Department of Psychiatry, University of Toronto, Toronto, Canada; 6 Maryland Psychiatric Research Center, Department of Psychiatry, University of Maryland School of Medicine, Baltimore, MD USA

**Keywords:** Schizophrenia, Biomarkers

## Abstract

Functional impairments contribute to poor quality of life in schizophrenia spectrum disorders (SSD). We sought to (Objective *I*) define the main functional phenotypes in SSD, then (Objective *II*) identify key biopsychosocial correlates, emphasizing interpretable data-driven methods. *Objective I* was tested on independent samples: *Dataset I* (*N* = 282) and *Dataset II* (*N* = 317), with SSD participants who underwent assessment of multiple functioning areas. Participants were clustered based on functioning. *Objective II* was evaluated in *Dataset I* by identifying key features for classifying functional phenotype clusters from among 65 sociodemographic, psychological, clinical, cognitive, and brain volume measures. Findings were replicated across latent discriminant analyses (LDA) and one-vs.-rest binomial regularized regressions to identify key predictors. We identified three clusters of participants in each dataset, demonstrating replicable functional phenotypes: *Cluster 1*—poor functioning across domains; *Cluster 2*—impaired *Role Functioning*, but partially preserved *Independent* and *Social Functioning*; *Cluster 3*—good functioning across domains. Key correlates were *Avolition, anhedonia, left hippocampal volume*, and measures of emotional intelligence and subjective social experience. *Avolition* appeared more closely tied to *role functioning*, and *anhedonia to independent and social functioning*. Thus, we found three replicable functional phenotypes with evidence that recovery may not be uniform across domains. *Avolition* and *anhedonia* were both critical but played different roles for different functional domains. It may be important to identify critical functional areas for individual patients and target interventions accordingly.

## Introduction

Functional impairment is recognized as a major deleterious consequence of schizophrenia spectrum disorders (SSD), and is distinct from symptom severity^1,2^. Functional impairment can affect interpersonal relationships, ability to pursue constructive activities, meeting role expectations, and functional capacity for independent living^3–5^. In general, SSD negatively impacts functioning in these areas, but outcomes are heterogeneous. A small but definite proportion (13–15%) of individuals affected by SSD achieve good social functioning^6^, comparable to a never-psychotic comparison group^7^. However, the majority of individuals experience intermediate outcomes, while another proportion experience severe impairment and profound disability^6,7^. Subjective outlook also ranges from optimism and hope to acceptance and resignation to despair^8^. Here, we use ‘functioning’ to refer to the individual’s degree and quality of engagement in the activities of daily life, ranging across areas such as occupation, education, social relationships and interactions, leisure pursuits, etc.

A plethora of biopsychosocial factors have been linked with functioning in SSD. Negative symptoms—especially avolition—are repeatedly identified as key correlates and predictors of poor functioning^9–11^. The same is true for communication abnormalities^12^. Meanwhile, a shorter duration of untreated psychosis has been related to better outcomes^13^. A range of neuroimaging findings related to functional recovery include frontal-limbic and whole-brain volumes, ventricular volumes, fractional anisotropy of the inferior longitudinal and arcuate fasciculi, and task-based activation of brain networks (especially social cognition networks)^14,15^. Functioning has also been associated with performance across wide-ranging neurocognitive domains, including processing speed, attention, memory, reasoning, and verbal ability^16,17^. Alongside general neurocognition, social cognition has demonstrated particularly strong relationships to functioning in SSD^9,18,19^. Relationships with cognition stretch across various domains of functioning^20^ can also be observed longitudinally^21^, and remain even when accounting for symptom severity^16^. Subjective cognitive empathy (the ability to understand the perspectives of others has also been related. Sociodemographic factors predicting better functioning include higher education, work history, and female sex^13^^,^^22^.

Previous studies examining the correlates of functional impairment in SSD have revealed a complex multifactorial landscape. Several network analyses have been conducted in large samples (*N* = 408–2022)^23–26^. The results have been fairly consistent, demonstrating clusters of intercorrelations among functional domains and among cognitive tests, with social cognition somewhat separated from other cognitive domains. There were also prominent connections between different areas of functioning and negative symptoms, as well as between functioning and cognition. Thus far, brain imaging findings have not been included in these data-driven approaches. Others have also demonstrated the complexity of examining potential cognitive determinants of functional outcomes in SSD. Overall cognition and processing speed predicted social and occupational functioning in one study, but the effect was no longer significant when accounting for negative symptoms^27^. Similarly, the relationship between neurocognition and functioning appears to be mediated through social cognition^28^.

Our goal was to parse the complexity of the interrelationships among functioning and relevant biopsychosocial factors in order to derive a concise and clinically actionable understanding of functional phenotypes in SSD. Emphasis was placed on using interpretable, data-driven methods, and on rigorously cross-validating the findings to generate reproducible results. To this end, our first objective (I) was to define the main functional phenotypes in clinically stable outpatients with SSD: i.e., what do individuals tend to experience? This was carried out by clustering participants and identifying principal components of functioning. Validation was carried out in an independent sample. Our second objective (II) was to identify the most important biopsychosocial correlates of functional phenotype in SSD: i.e., which patient characteristics, when taken together, are most indicative of an individual’s functional phenotype? This was done with a machine learning approach using latent discriminant analysis (LDA) because this approach allowed for the selection of key predictors and provided information on the strength and direction of predictor loadings while accounting for higher-order interaction effects. Findings were validated by using an out-of-sample test set and by comparing results among different analytical approaches.

## Methods

### Participants

All participants were clinically stable adult outpatients with schizophrenia spectrum disorder (SSD) (Table 1) and provided written informed consent; all study protocols were approved by relevant review boards.

**Table 1:** Participant characteristics.

Variable	Dataset I	Dataset II	*p*	Cohen’s *D*
*n*	282	317		
Age—years (SD)	32.2 (10.2)	43.3 (10.1)	<0.001	1.09
Sex—*n* Female (%)	97 (34.4%)	87 (27.4%)	0.07	
Diagnosis—*n* (%)			<0.001	
Schizophrenia	206 (73.0%)	270 (85.2%)	<0.001	
Schizoaffective disorder	54 (19.1%)	47 (14.8%)	0.19	
Schizophreniform disorder	7 (2.5%)	0 (0%)	0.005	
Psychosis NOS	15 (5.3%)	0 (0%)	<0.001	
BPRS total score (SD)	30.9 (7.7)	31.1 (7.8)	0.68	0.03
SANS global total (SD)	7.7 (3.3)	8.4 (3.1)	0.004	0.24

*Note*: Reported characteristics were based on participant report / self-identification. Race was tabulated differently for the two datasets: *Dataset I* (SPINS)—Asian *n* = 42 (15%); Black *n* = 95 (34%); White *n* = 145 (51%); Mixed and other races *n* = 18 (6%). Ethnicity: Hispanic/LatinX *n* = 33 (12%). *Dataset II*—Black *n* = 130 (41%); White *n* = 164 (52%); Other race *n* = 21 (7%). *SD* standard deviation, *BPRS* brief psychiatric rating scale (18 Item), *SANS* scale for the assessment of negative symptoms. *P* values shown are either *t*-tests for continuous variables or Fisher’s Exact tests for categorical variables.

*Dataset I* (*N* = 282) was used for both *Objectives I* & *II*. Participants were drawn from the multi-site social processes initiative in the neurobiology of schizophrenia(s) (SPINS)^28^ and underwent the full range of assessments below. Recruitment took place at the Zucker Hillside Hospital (Glen Oaks, NY), the Centre for Addiction and Mental Health (Toronto, Ont.), and the Maryland Psychiatric Research Center (Baltimore, MD). The assessments were conducted across three visits (MRI, neurocognition, social cognition, clinical assessments, and participant self-reports). For these analyses, we selected SSD participants who had completed assessments for functioning. SSD participants met the Diagnostic and Statistical Manual of Mental Disorders, 5th Edition (DSM-5) criteria for schizophrenia, schizoaffective disorder, schizophreniform disorder, or unspecified psychotic disorder. Other aspects of this cohort, along with further details about the recruitment, ascertainment, and assessments, have been previously described by Oliver et al.^28^, Hawco et al.^29^, and Tang et al.^30^.

*Dataset II* (*N* = 317) was a validation set for *Objective I* and underwent functional outcomes assessments. Imaging and social cognitive phenotyping were not available, so this Dataset was not included in *Objective II*. Participants were recruited primarily from the Zucker Hillside Hospital, with adjunctive recruitment conducted at the Manhattan Psychiatric Center. For these analyses, we selected SSD participants who had complete functional outcomes assessments, and who did not re-enroll in the SPINS study. SSD participants met DSM-IV-TR criteria for either schizophrenia or schizoaffective disorder. An interim analysis from this dataset, along with further details about the ascertainment and assessments, have been described by Shamsi et al.^31^.

### Assessment of functioning

For *Dataset I*, functioning was assessed with the Birchwood social functioning scale (*BSFS*)^3^ and quality of life scale (*QoL*)^5^, both clinician-rated scales based on participant reports. Each subscale was considered separately.

For *Dataset II*, related functioning domains were assessed, though with different scales and modalities. We used the following items: work and interests from the Hamilton rating scale for depression (*Ham-D*; clinician-rated)^32^, role and residential functioning from the multidimensional scale of independent functioning (*MSIF*; clinician-rated)^4^, leisure activities, social frequency, and degree of social activity from social adjustment scale (*SAS*; self-report)^33^, and financial and communication skills from performance-based skills assessment (*UPSA*; performance-based)^4^. Table 2 and the Supplemental Methods include further details.

**Table 2:** Overview of functioning measures.

A—Dataset I	B—Dataset II
Birchwood Social Functioning Scale (*BSFS*)	Hamilton Rating Scale for Depression (*Ham-D*)
Social Engagement Withdrawal	Work and Interests
Interpersonal Communication/Relationships	
Prosocial Activities	Multidimensional Scale of Independent Functioning (*MSIF*)
Recreation	Global Role Functioning
Independence—Performance	Global Residential Functioning
Independence—Competence	
Occupation/Employment	Social Adjustment Scale—Self Report (*SAS*)
	Leisure Activities
Quality of Life Scale (*QoL*)	Social Frequency
Interpersonal Relationships	Degree of Social Activity
Instrumental Role	
Intrapsychic Foundations	UCSD Performance-Based Skills Assessment (*UPSA*), abbreviated version
Common Objects & Activities	
	Financial Skills
	Communication Skills

Subscales and items from the following measures were used for *Dataset I* (sub-table A) and *II* (sub-table B) to define the functioning phenotypes and principal components of functioning. Additional details are presenting in the Supplemental Methods.

### Assessment of biopsychosocial measures

Biopsychosocial measures were evaluated as correlates of functional phenotype for *Objective II* (*Dataset I*). Detailed descriptions are listed in Supplemental Methods and Table 3.

**Table 3:** An overview of biopsychosocial predictors.

DEMO: sociodemographic and personal characteristics	COG: general neurocognition (*MATRICS*)	SCOG: social cognition
Sex	Processing Speed	MSCEIT
Age	Attention and Vigilance	*ER40* Accuracy
Ethnicity: Hispanic	Working Memory	*ER40* Speed
Race: White	Verbal Learning	*TASIT* Total Score
Race: Black	Visual Learning	*RMET* Total Score
Race: Asian	Reasoning and Prob. Solving	*RAD* Total Score
English as Primary Language	*WTAR* Standard Score	
Family Hx SSD		*MRI*: Structural Brain Imaging
Parental Educational Attainment	*SYMP*: Clinical Ratings of Psychosis Symptoms	Total Ventricular Volume
Age of Onset		Total Brain Volume
Duration of Illness	*BPRS* Anxiety and Depression	Total Gray Matter Volume
	*BPRS* Positive Symptoms	R/L Superior Temporal Volume
*SELF*: Subjective Psychological Experiences	*BPRS* Activation	R/L Entorhinal Volume
	*BPRS* Hostility	R/L Hippocampal Volume
*IRI* Perspective Taking	*SANS* Affective Flattening	R/L Amygdala Volume
*IRI* Fantasy	*SANS* Alogia	R/L Thalamic Volume
*IRI* Empathic Concern	*SANS* Avolition	R/L Superior Frontal Volume
*IRI* Personal Distress	*SANS* Anhedonia	R/L Caudal Middle Frontal Volume
*SPQ* Cognitive and Perceptual		R/L Lateral Orbitofrontal Volume
*SPQ* Interpersonal		R/L Medial Orbitofrontal Volume
*SPQ* Disorganized		R/L Rostral Middle Frontal Volume
		R/L Inferior Frontal Volume
		Mean Prefrontal Cortical Volume

The following 65 variables were used in the linear discriminant analysis to predict functional phenotype. An overview of the assessment scales can be found in Supplement Section B.

#### Sociodemographic and personal characteristics

We used participant report and electronic health records (EHR) to determine self-identified demographic information and personal characteristics potentially relevant for functional outcomes, including *family history of SSD*, *English as primary language, parental educational attainment* (highest known), and *duration of illness* for SSD.

#### Assessment of Biopsychosocial MeasuresClinical Symptoms

Psychosis symptoms were assessed using the Brief Psychiatric Rating Scale (*BPRS*)^34^ and the Scale for Assessment of Negative Symptoms (*SANS*)^35^. Subscale scores were used to represent different symptom domains.

#### General neurocognition

General neurocognition was assessed with the NIMH-measurement and treatment research to improve cognition in schizophrenia (*MATRICS*) consensus battery^36^. (The Mayer–Salovey–Caruso emotional intelligence test (*MSCEIT*) was categorized with the social cognition measures)^37^. T-scores for each domain, i.e., processing speed, attention and vigilance, working memory, verbal learning, visual learning, and reasoning/problem-solving, were used.

#### Social cognition

Emotional intelligence, emotion processing, mental state attribution, and social perception were assessed with: the *MSCEIT*; the Penn emotion recognition 40 (*ER40*)^38^; the awareness of social inference test-revised (*TASIT*)^39^; the reading the mind in the eyes task (*RMET*)^40^; and relationship across domains (*RAD*)^41^. The assessments were chosen to cover a range of social cognitive domains because of their inclusion in the social cognition psychometric evaluation (*SCOPE*)^42^.

#### Subjective psychological experiences

Self-report questionnaires assessed subjective experiences of interpersonal situations with the interpersonal reactivity index (*IRI*)^43^ and the schizotypal personality questionnaire-brief version (*SPQ-B*)^44^.

#### MRI

Brain volume measures representing replicable structural MRI findings in schizophrenia^45^ were included as potential predictors (Table 3). The rationale was that key biological signals associated with SSD diagnosis may converge on the level of brain structure, and may be associated with functional phenotypes in SSD. That is, individuals with higher biological loading for schizophrenia may show more pronounced differences in brain structure as well as greater functional impairment. Magnetic Resonance Imaging (MRI) was performed on six 3 T scanners across the 3 sites and harmonized as previously described by Oliver et al.^46,47^. Imaging parameters and additional details can be found in the Supplemental Methods. T1-weighted anatomical images were corrected for intensity non-uniformity (INU) with N4BiasFieldCorrection^48^, distributed with ANTs 2.2.0 (RRID:SCR_004757^49^). Brain surfaces were reconstructed and subcortical volumes were calculated using Freesurfer recon-all (FreeSurfer 6.0.1, RRID:SCR_001847^50^). The selected volumetric measures (Table 3) represent replicable structural MRI findings in schizophrenia Right and left hemisphere measures were included separately.

### Objective I: defining functional phenotypes

**Fig. 1:**
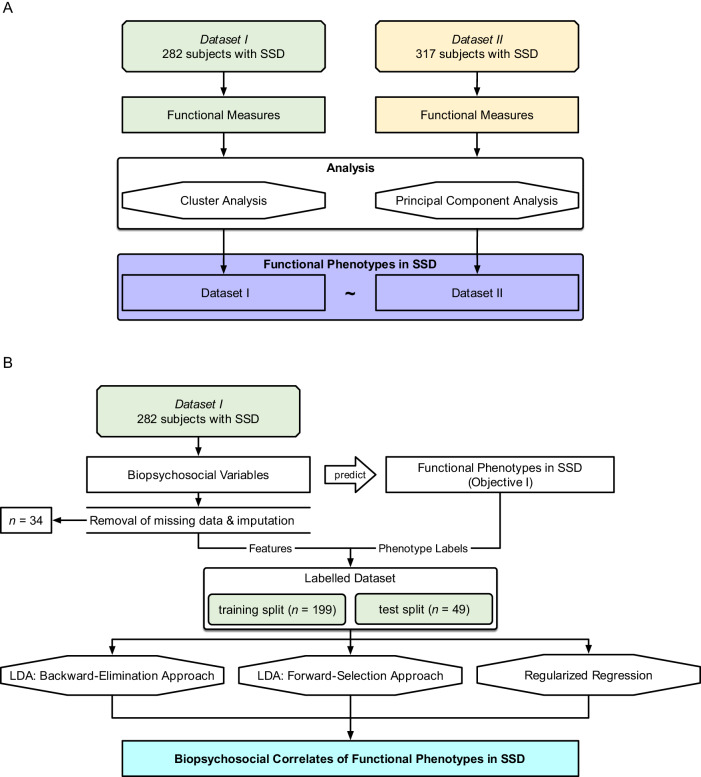
Overview of analytical approaches. **A** *Objective I*: Defining functional phenotypes. Two datasets (${N}_{I}=282$, ${N}_{{II}}=317$) were analyzed separately, and unsupervised clustering and principal component analysis were performed on individual functioning items to define 3 functioning phenotypes. **B** *Objective II*: Predicting functioning phenotypes (*Objective I*) based on demographics, neurocognition, social cognition, and brain structure. Thirty-four participants were excluded from *Dataset I* due to insufficient data; resulting in $n=248$ remaining participants. We performed three methods (backward-elimination LDA, forward-selection LDA, and regularized regression).

*Objective I* was tested on both datasets, with *Dataset II* as an independent validation sample (R packages listed in Supplemental Table 1). The aim was to define functional phenotypes based on patterns in the expression of individual functioning measures (Table 2) using an unsupervised clustering approach with bootstrapping. This approach was chosen because, assuming that the samples are representative of the larger SSD population, clustering informs us about the functional phenotype patterns which we might expect to observe among patients in a clinical setting. Additionally, a principal component analysis was performed to describe functioning domains and aid interpretations of the functional phenotypes. The analysis pipeline is shown in Fig. 1A.

#### Cluster analysis

**Fig. 2:**
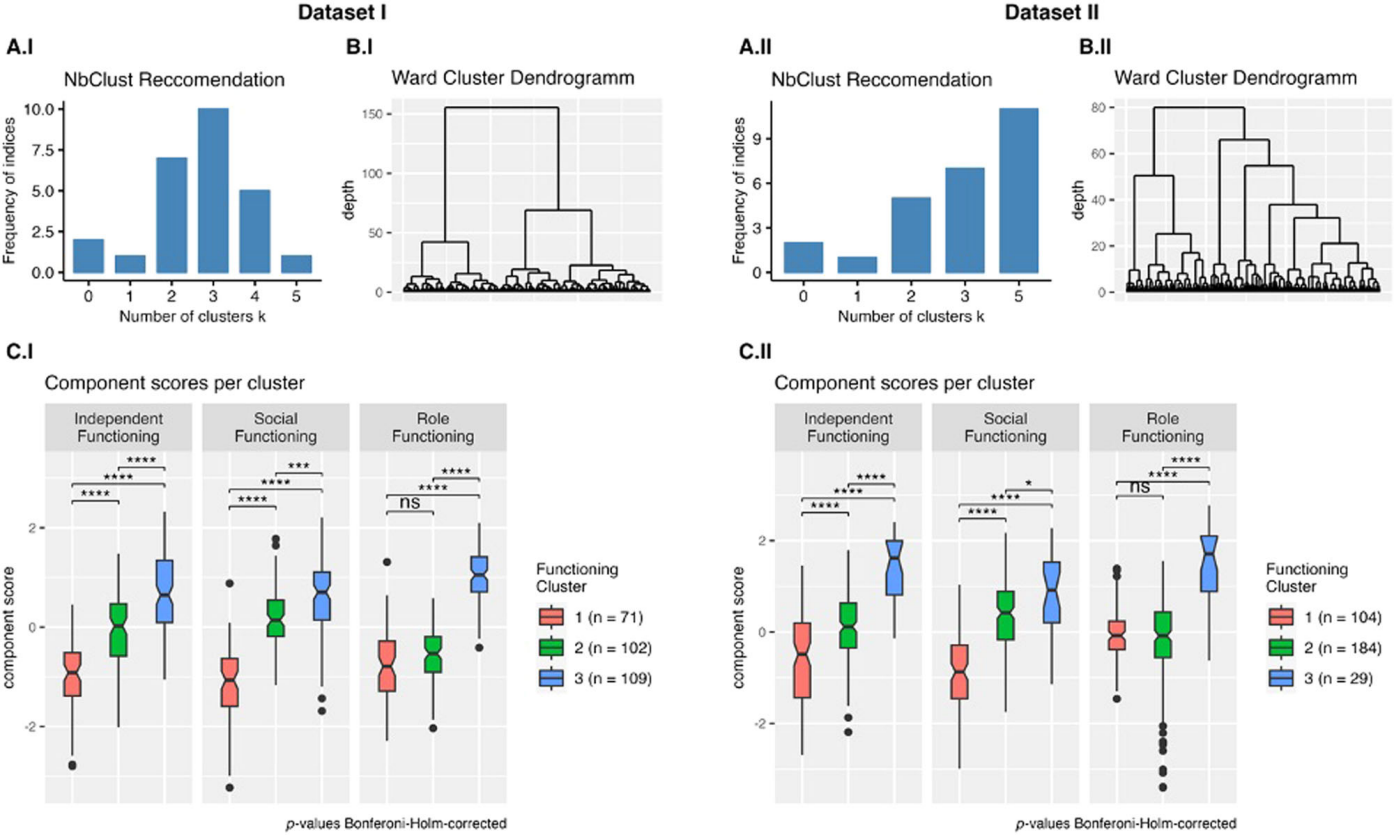
Summary of functional outcomes clusters for *Dataset I* and *Dataset II.* Subplots A.I and A.II depict the frequency of *NbClust* provided indices that recommended a certain cluster size for each dataset. Subplots B.I and B.II show the hierarchical clusters produced by the Ward algorithm (applying a Euclidean distance as a distance metric). Subplots C.I and C.II compare the functional phenotype clusters based on the functional components resulting from the PCA. Higher component scores reflect better functioning in that domain. Pairwise comparisons ($t$-tests) were used to evaluate statistical significance (Bonferroni–Holm-corrected^52^: ns not significant, *p < 0.05, **p < 0.01, ***p < 0.001, ****p < 0.0001).

We performed bootstrapped hierarchical Ward clustering across the individual functional items^51^, optimizing Euclidean distance. The optimal number of clusters (*n* = 3) was determined using the *NbClust* R package in Dataset I based on 11 functioning items (Fig. 2). For *Dataset II*, clustering was conducted on 8 functioning items. From *NbClust*, 11 of the metrics proposed $k=5$ clusters as the optimal cluster number; the runner-up was $k=3$ with 7 indices suggesting this as the optimal cluster number. For consistency, 3 cluster solutions were produced for both datasets. Bootstrapping was performed 100 times in each sample using the *clusterboot* function from the fpc *R package* to determine optimal clustering and cluster stability. To compare functioning and biopsychosocial variables among the three clusters, we used pairwise *t*-tests with Bonferroni–Holm-corrected *p*-values^52^. Group effects for demographic variables were evaluated using ANOVA for age and clinical ratings and Fisher’s Exact test for sex, race, and diagnosis. The generalizability and stability of the clusters were established by running the analyses on independent samples, using different functioning items; resampling was not employed.

#### Principal component analysis

To aid interpretability, principal component analysis (PCA)^53^ was performed on the individual functioning measures. The scree plot was visually inspected, and Kaiser’s criterion was used to determine the optimal number of components (Supplemental Fig. 1). For both datasets, we used a three-component solution with Promax rotation.

### Objective II: Identifying Biopsychosocial Correlates

*Objective II* was tested on *Dataset I*. The aim was to identify the subset of biopsychosocial correlates which, when taken together, most accurately classify participants into the 3 functional phenotypes defined in *Objective I*. We evaluated 65 intercorrelated biopsychosocial variables including sociodemographic and personal characteristics, psychosis symptom ratings, general neurocognition, social cognition, and structural brain imaging metrics (Table 3). The emphasis was on understanding how different combinations of biopsychosocial correlates may be related to functioning, and not on building a classification model per se. The analysis pipeline is shown in Fig. 1B. Variables which may not have a main effect on functional phenotype were nevertheless included because of the possibility of secondary interactions with other variables.

#### Preprocessing

Due to missing values in biopsychosocial measurements, we implemented an exclusion-imputation strategy. We removed 34 individuals from *Dataset I* who had 4 or more measures missing (5% of the total feature set). For individuals with 1–3 missing measures ($n$ = 28), we imputed these using the *mice* R package and predictive mean matching, resulting in a total sample size of 248 individuals from *Dataset I*. A total of 40 observations were imputed out of over 16,000 (0.2%). After imputation, an 80/20 train-test split was made. In order to normalize coefficients and avoid bias from the test set, each of the 65 predictors was standardized by calculating *z*-scores with respect to the training split. Sample characteristics for both the train and test set are shown in Supplemental Table 2.

#### Latent discriminant analysis (LDA) classification

We selected linear discriminant analysis (LDA) as our classification algorithm due to a) its ability to perform multi-class classification suitable for the 3 functional phenotypes and b) its interpretability and ability to provide variable coefficients (i.e., linear discriminants; LD) that determine the strength and directionality (i.e., positive or negative) of the contributing predictor. Two LDs were examined because LDAs are limited to a dimensional space lower than the number of groups being classified (3 clusters—1 = 2 LDs). The LDA function from the *MASS* R package was used. The training was done on an 80% training set using leave-one-out cross-validation. The generalizability of the resulting model was determined on a 20% set-aside test set. The whole dataset was used for reporting the final LD coefficients. The target metric for the classification was accuracy: i.e., the percentage of correct classifications.

#### Backward-elimination linear discriminant analysis

The aim was to identify an interpretable set of key correlates out of the 65 biopsychosocial predictors (Table 3) that best describe the functional phenotype. A limit of up to 10 variables was defined a priori. Ideally, we would evaluate all possible combinations of variables at each level from 1 to 10 (e.g., level 4 would test all combinations of *k* = 4 variables out of the *n* = 65 possible predictors). However, trying every combination of $k=10$ variables for $n=65$ total predictors would result in almost 180 billion combinations (see Supplemental Table 3). A feasible computational boundary was, therefore, set at 2 million variable combinations. To keep the number of combinations below this threshold at each iteration, we applied a stepwise elimination (i.e., backwards elimination) of predictors that contributed least to the prediction performance in the previous step, i.e., lowest average test-set accuracy.

The variable selection proceeded as follows: if the number of combinations in a given level exceeded the computational threshold, we eliminated poor predictors until the threshold was met. Poor predictors were defined as the predictors with the lowest maximum accuracy in the previous level. An overview of the eliminated variables and combination counts is provided in Supplemental Table 4. For example, at level 5, 16 variables were eliminated in order to stay within the computational threshold; so, we selected all *k* = 5 combinations from *n* = 49 variables and ran a total of 1,906,884 LDA models. This was continued until we reached level 10 consisting of 10 predictors.

#### Forward-selection linear discriminant analysis

With the aim of identifying an interpretable set of key correlates without a pre-defined limit to the total number, we developed an approach using a successive forward selection of predictors and a natural, data-driven stopping point. For each iteration, the best combinations of one, two, three, and four variables were identified—due to these being within our computational boundary of 2 million (Supplemental Table 3). A predictor was selected (i.e., “*fixed”*) if it appeared in at least 3 of 4 best performing models (based on test-set accuracy), allowing for interaction effects where a predictor may be valuable in combination with other predictors, but not on its own. Selected predictors were added iteratively to the fixed predictor set and included in all subsequent levels. This process terminated when no further consistent variables were found.

More specifically, for the first iteration, we evaluate all 1–4-variable combinations of the 65 predictor variables from Table 3. Two predictors appeared in 3 or more of the best-performing models (*Avolition* and *Anhedonia*). These were then fixed as predictors, and included in all subsequent iterations. For the second iteration, we tested *Avolition* and *Anhedonia* in combination with all 1–4-variable combinations of the remaining 63 predictor variables. This time, *Left Hippocampal Volume* was selected as a fixed predictor. For the third iteration, we tested *Avolition*, *Anhedonia*, and *Left Hippocampal Volume* in combination with all 1–4 variable combinations of the remaining 62 predictor variables, and so forth. Variables were added over 4 iterations. For the fifth iteration, none of the remaining variables appeared in 3 or more of the best-performing models, so the process reached its natural termination.

#### Regularized regression

To validate the findings from the LDAs, we used the L1 regularized regression, i.e., least absolute shrinkage and selection operator (LASSO) using the R *glmnet* package as a penalizing regression that performs variable selection and prediction in one step^54^. However, this regression functions as a binary classification and cannot be directly applied to a three-class problem. Thus, we applied a one-vs-rest strategy—computing two models that were analogous to the two latent discriminants from the LDAs described above. The appropriate model $\lambda$ hyperparameter was determined using the minimum mean cross-validation error. We report the archived accuracies on the training and test sets, as well as coefficients for the non-penalized predictors. These coefficients were determined using an L2-regularized regression, i.e., ridge regression, trained on the entire dataset using the L1-selected predictors to allow for the retainment of the selected predictors.

Of note, the regression was used primarily to validate findings from the LDAs, and is limited by its inability to classify all three clusters in the same model. The 2-class prediction accuracies reported for the LASSO one-vs-rest should be interpreted on a difference scale from the 3-class prediction accuracies performed with the LDA because the random-guessing accuracy is 50% for a balanced two-class prediction problem, and 33% for a 3-class prediction.

#### Constructing a final model

Predictors emerging consistently from both the backward-elimination and forward-selection LDAs (the primary methods) were identified as replicable key correlates of functional phenotype. The key correlates were used as predictors for a final LDA model describing the full sample to provide a unified summary of the LDA results. We recorded the confusion matrix of the final model as well as accuracy and balanced accuracy—defined as the average of the recall of each of the three classes.

## Results

### Objective I: defining functional phenotypes

#### Functional phenotype clusters

Participants in each Dataset were clustered according to functioning measures. Based on the *NbClust* package (Fig. 2A), we chose $k=3$ clusters for both datasets. Bootstrapping resulted in mean Jaccard similarity (degree of overlap) of 0.66 for *Cluster 1*, 0.58 for *Cluster 2*, and 0.75 for *Cluster 3* in *Dataset I*; and mean Jaccard similarity of 0.57, 0.66, and 0.65, respectively, in *Dataset II*. Supplemental Fig. 2 shows how individual functioning measures were distributed across the 3 clusters in each Dataset. Generally, participants in *Cluster 1* reported poor functioning, while participants in *Cluster 3* reported better functioning. Those in *Cluster 2* were largely intermediate but reported higher levels of social engagement, interpersonal communication and interpersonal relationships, and social frequency similar to *Cluster 3*; on the other hand, participants in *Cluster 2* reported poorer functioning for occupation/employment, instrumental role, work and interests, and global role functioning, similar to *Cluster 1*.

#### Principal components of functioning

Principal component analysis (PCA) was used to simplify the functioning measures and better illustrate the differences among the 3 functional phenotypes. The PCA suggested similar three-component solutions for both datasets (Table 4) where the components could be described as representing *independent functioning* (skills and activities related to functioning independently), *social functioning* (depth and degree of interpersonal relationships), and role functio*ning* (engagement in occupational and instrumental role activities).

**Table 4:** PCA loadings for dataset I (sub-table A) and II (sub-table B).

Scale: Item *(Description/Examples)*	Independent Functioning	Social Functioning	Role Functioning
*A—Dataset I*			
*BSFS: Recreation* (frequency; e.g., hobbies, exercise)	**0.91**		
*BSFS: Independence*—*Performance* (frequency of doing activities, e.g., chores, self-care)	**0.76**		
*BSFS: Prosocial Activities* (frequently participating in society, e.g., going to public places like movies and restaurants, playing sports)	**0.62**	0.40	
*QoL: Common Objects & Activities* (possession of common objects, e.g., wallet, library card; constructive activities, e.g., movies, shopping)	**0.60**		
*QoL: Intrapsychic* (internal drive, e.g., purpose, motivation, curiosity, engagement)	**0.38**	0.32	0.33
*BSFS: Independence—Competence* (comfort with activities, e.g., chores, self-care)	**0.34**		
*BSFS: Interpersonal Communication/Relationships* (number and supportiveness of relationships)		**0.89**	
*QoL: Interpersonal Relationships* (number and depth of relationships)		**0.69**	
*BSFS: Social Engagement Withdrawal* (time spent interacting vs. alone)		**0.63**	
*BSFS: Occupation/Employment* (gainful employment or homemaking)			**0.93**
*QoL: Instrumental Role* (gainful employment, homemaking or educational pursuits)	0.31		**0.88**
*B - Dataset II*			
*UPSA: Financial Skills* (counting change and paying bills)	**0.85**		
*UPSA: Communication Skills* (making telephone calls)	**0.84**		
*MSIF: Global Residential Functioning* (living independently, doing chores)	**0.59**		
*SAS: Degree of Social Activity* (active initiation)		**0.91**	
*SAS: Social Frequency* (frequency of activities)		**0.87**	
*Ham-D: Work and Interests* (attitude and ability to sustain work)			**0.87**
*SAS: Leisure Activities* (interest in constructive pursuits)			**0.47**
*MSIF: Global Role Functioning* (employment, caretaking or educational pursuits)		0.32	**0.41**

The table shows the Promax rotated component loadings (loadings < 0.3 are masked). The first component, *independent functioning*, explained $40.6{\boldsymbol{ \% }}$ and $27.3{\boldsymbol{ \% }}$ of the variance in *Dataset I* and *II* respectively. It was characterized by recreational activities, financial skills, and residential functioning. The component *social functioning* explained a variance of $11.2{\boldsymbol{ \% }}$ and $16.7{\boldsymbol{ \% }}$ and grouped items around social and interpersonal activities. And *role functioning* explained $9.0{\boldsymbol{ \% }}$ and $13.2{\boldsymbol{ \% }}$ of total variance and summarized items of employment and role. Primary loadings are bolded.

#### Summary of functional phenotypes

Three functional phenotypes were defined by comparing the principal components of functioning across the three clusters of participants in each Dataset (Fig. 2C, pairwise comparisons in Supplemental Table 5). *Cluster 1* represents an impaired phenotype with low functioning in all three domains (Prevalence: *Dataset I*—25%; *Dataset II*—33%). *Cluster 2* represents an intermediate phenotype with impaired *Role Functioning* similar to that of *Cluster 1*, but partially preserved *Independent* and *Social Functioning*. *Cluster 3* represents a resilient phenotype with higher *Independent, Social*, and *Role Functioning* than both other clusters (Prevalence: *Dataset I*—39%; *Dataset II* 9%). *Cluster 2* is the most prevalent phenotype in *Dataset II* (58%) while representing 36% of individuals in *Dataset I*. Supplemental Table 6 describes demographic and clinical characteristics; there was no effect of the cluster on age, sex, or diagnosis for either dataset, but there was an interaction between cluster and total SANS score for both datasets and for total BPRS score in *Dataset I* and race in *Dataset II*.

### Objective II: biopsychosocial correlates of functional phenotypes

#### Backward-elimination LDA

**Fig. 3:**
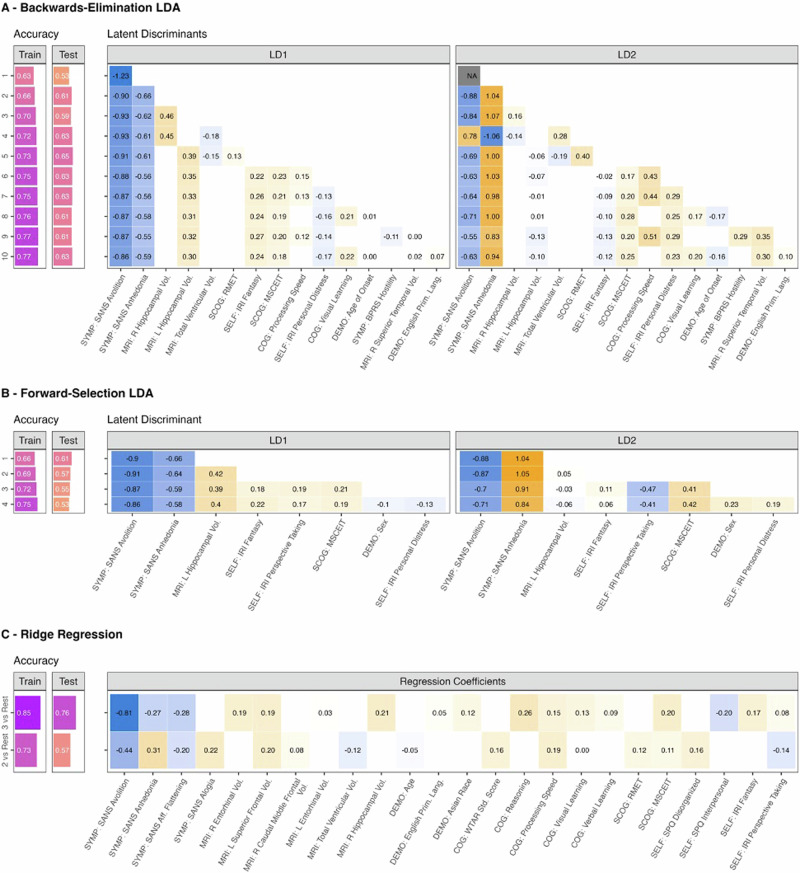
Overview of predictor variables of functioning phenotype. Best performing models are described for the **A** Backward-Elimination LDA Approach, **B** Forward-Selection LDA approach, and **C** Ridge Regression, with the iteration step labeled on the y-axes for the LDA models (**A**, **B**) and the model targets labeled for the Ridge regression (**C**). Model accuracies, i.e., the frequency at which the model correctly categorizes each participant into the correct cluster, are shown on the left for the training/test sets; darker colors indicate higher accuracy. Predictors for each of the models are shown across the *x*-axes of the heatmaps on the right, with coefficients plotted for each iteration of the models (darker blue indicates more negative loadings, brighter orange indicates more positive loadings). Note that *Avolition*, *Anhedonia*, and *Left Hippocampal Volume* appear to be the most consistent predictors of functional phenotype in (**A**), and *Avolition* and *Anhedonia* show the best generalization performance (accuracy of 0.61 on the test set).

**Fig. 4:**
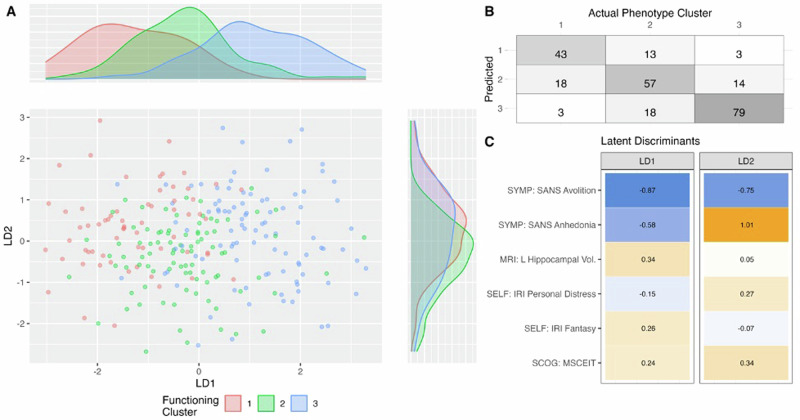
Objective II results: final LDA model with key biopsychosocial correlates. The most consistent predictors were identified as *Avolition*, *Anhedonia*, *Left Hippocampal Volume*, *IRI Personal Distress, IRI Fantasy*, and *MSCEIT*. **A** The Linear Discriminants (LDs) were trained on the full dataset values are plotted for each functioning phenotype cluster. LD1 separates the 3 classes from each other in a graded manner, with the highest values in the highest functioning cluster, followed by the intermediate cluster, and with the lowest values in the lowest functioning cluster. LD2 separates *Cluster 2* from *Clusters 1* and *3*, with higher, similar values for both *Cluster 1* and *Cluster 3*, but lower values for *Cluster 2*. **B** Confusion matrix between actual and predicted clusters when predicting on the full dataset using the final model. The accuracy is 0.72, and the balanced accuracy—defined as the average of recalls—is 0.71. **C** LD1 and LD2 coefficients for the final model.

Several predictors were selected consistently by the backward-selection LDA for classifying functional phenotypes in *Dataset I* (Fig. 3A). *Avolition* and *Anhedonia* were the first and second predictors selected, and they remained consistent in each of the 10 levels. *Hippocampal Volume*, either right or left, appeared at the third level and also remained consistent throughout. Of note, *right and left hippocampal volume* are highly correlated, with $r=0.84$ (Pearson coefficient; $p \,<\, 0.001$). Other consistent predictors included the *Fantasy* and *Personal Distress* subscales from the IRI, the *MSCEIT* (a measure of emotional intelligence) and *Processing Speed* portions of the *MATRICS*. *Latent Discriminant 1 (LD1)* separated the clusters along the overall level of functioning, with the highest value in *Cluster* 3, followed by *Cluster* 2, then *Cluster 1* (Fig. 4A)*. LD2* separated the other clusters from *Cluster 2*. Of note, *Anhedonia, IRI Fantasy* and *Personal Distress* show opposite directionality in their loadings for *LD1* vs. *LD2*. *Hippocampal Volume* was highly loaded on *LD1* but not *LD2*. Peak training accuracy was 77% (levels 9 and 10), and peak test set accuracy was 65% (level 5).

#### Forward-selection LDA

Eight predictors were identified by the forward-selection LDA (Fig. 3B). As in the backward-selection approach, *Avolition, Anhedonia, Left Hippocampal Volume, MSCEIT* score*, IRI Fantasy* and *IRI Personal Distress* were identified as key predictors of functional phenotype. Additionally, *IRI Perspective Taking* and *Sex* were also selected. The training and test accuracies of the fourth (and final) level of the forward-selection model were 75% and 53% respectively. Of note, similar to the backward LDA, both *Anhedonia* and *Personal Distress* loaded in opposite directions for LD1 vs. LD2, and *Left Hippocampal Volume* was loaded primarily on LD1.

#### Regularized regression analyses

Regularized regression models separately classified *Cluster 3*-vs.-rest (roughly analogous to LD1), and *Cluster 2*-vs.-rest (roughly analogous to LD2). Results largely substantiated the LDA findings (Fig. 3C). Most of the consistent predictors identified in the LDAs were also selected by the LASSO models: *Avolition*, *Anhedonia*, *Hippocampal Volume (Right)*, *MSCEIT* score, and *IRI Fantasy*. *IRI Personal Distress* was not selected. Loadings largely reflected the patterns found in the LDAs, with *Avolition* loaded negatively on both models, but *Anhedonia* loading negatively on the *Cluster 3*-vs.-rest model and positively on the *Cluster 2-*vs.-rest model. *Right Hippocampal Volume* was selected on the *Cluster 3*-vs.-rest model but not on the *Cluster 2-*vs.-rest model. Training and test accuracies were 83% and 78% for *Cluster 3*-vs.-rest, and 67% and 66% for *Cluster 2*-vs.-rest.

#### Summary of biopsychosocial correlates

Six predictors were identified as replicable key correlates of functional phenotype: *Avolition, Anhedonia, Left Hippocampal Volume, IRI Personal Distress, IRI Fantasy*, and *MSCEIT* score. Figure 4 illustrates the final model performance with 72% accuracy and 71% balanced accuracy, with most misclassifications occurring for adjacent clusters.

**Fig. 5:**
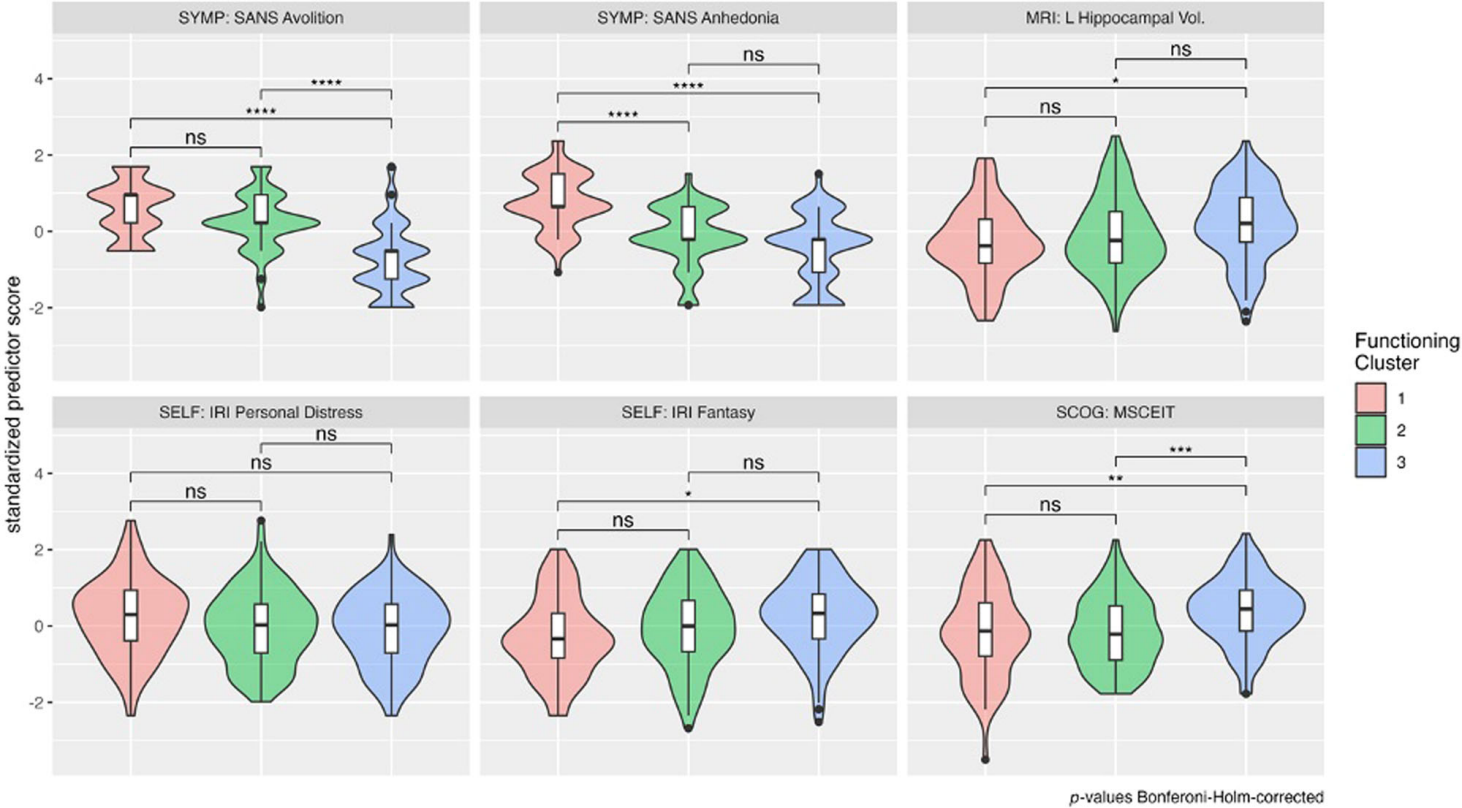
Final predictors of functional phenotype clusters. Standardized scores for each of the final predictors (*Objective II*) are shown for each of the 3 functional phenotypes (*Objective I)*. After visual inspection of histograms and check of normality, *t*-tests were performed. $P$-values for pairwise comparisons were adjusted using the Bonferroni–Holm method^52^ and reported as significant with $\alpha < 0.05$. Note: The functional phenotypes clusters are: Cluster 1—impaired phenotype across the social, independent, and role functional domains; Cluster 2—intermediate phenotype with impaired role functioning but partially preserved social and independent functioning; Cluster 3—resilient phenotype with higher role, independent and social functioning.

Figure 5 compares standardized scores for each variable across the functional phenotypes. Of note, *Cluster 2*, with poor *Role Functioning* but partially preserved *Independent* and *Social Functioning*, scored similarly to *Cluster 1* (impaired phenotype) in *Avolition*, and similarly to *Cluster 3* (resilient phenotype) in *Anhedonia*. Supplemental Table 7 details all predictors with significant group differences across the clusters.

## Discussion

Functional outcomes are critically important for individuals and families affected by SSD and demonstrate complex relationships with a range of biological, psychological, sociodemographic, clinical, and cognitive factors. A better understanding of functional phenotypes and their key biopsychosocial correlates is needed for prognosis and for identifying critical areas of intervention. Here, prioritizing both interpretability and reproducibility, we leveraged data-driven methods to define three main functional phenotypes in SSD, with six key biopsychosocial correlates. The functional phenotypes and domains were reproduced across two independent datasets, using different assessments for functioning (*Objective I*). Then, biopsychosocial correlates were consistently identified across multiple analytical strategies, each conducted with internal cross-validation and set-aside test samples (*Objective II*).

We identified three clusters of participants in each Dataset, demonstrating replicable functional phenotypes^1^: a relatively impaired phenotype (*Cluster 1)* with poor functioning in all three domains^2^; an intermediate phenotype (*Cluster 2)* with relatively impaired *Role Functioning* similar to *Cluster 1*, but partially preserved *Independent* and *Social Functioning*; and^3^ a resilient phenotype (*Cluster 3*) with good functioning in all three domains. A goal of this analysis was to identify clinically relevant characterizations of functioning in SSD—i.e., what types of patients are we likely to see from a functioning perspective? Therefore, these phenotypes are not intended to be biologically homogenous, and we did not attempt to separate primary illness effects, medication effects, etc. Because of the relatively large sample size and replication across independent samples, we propose that these three functional phenotypes may represent prominent patterns in functional outcomes among patients with SSD in outpatient treatment settings.

A substantial proportion of participants belonged to *Cluster 2* in both *Dataset I* (36%) and *Dataset II* (58%). This cluster identifies individuals who struggle with employment and other instrumental role activities (e.g., education, caretaking responsibilities) but maintain intermediate functioning in social relationships, skills for independent living, and pursuit of personal interests. The inverse pattern of relatively preserved role functioning but poor social and independent functioning *did not* emerge in our analyses and may not be a common outcome for patients with SSD. Delineating the *Cluster 2* phenotype is significant because it allows for the recognition of this outcome and the fact that functional outcomes can be uneven for a large proportion of individuals with SSD. The recognition of this phenotype is clinically important in itself because the recognition of patients’ strengths is vital to recovery-oriented care^55^. Without understanding or defining this phenotype, clinicians may assume that functional impairment is uniform and overlook important strengths that can be assets in the recovery process. In addition, it is possible that interventions should be targeted differently for patients with different functional phenotypes: those with impaired functioning across all areas may benefit most from interventions targeting *Social* and *Independent Functioning*, while *Role Functioning* should be emphasized for individuals with the intermediate phenotype. The differential patterns of impairment across these domains suggest that they may rely on different cognitive and/or biological substrates, and warrant further investigation to delineate these underlying processes and inform potential targeted treatment avenues.

From among 65 sociodemographic, cognitive, biological, and psychological features related to functioning, we identified six key correlates that were consistently selected for classification of functional phenotype: *Avolition, Anhedonia, Left Hippocampal Volume, MSCEIT* score, and the *IRI Fantasy* and *Personal Distress* subscales which measure, respectively, subjective ability to connect with fictional or imagined scenarios, and experience of troubling emotions during stressful situations. It is important to note that these features were not necessarily the most individually differentiated among the clusters, but rather they performed best and most consistently *in combination*—therefore there is a selection for features which are orthogonal to the others, adding the most unique information. The six key correlates identified here support a biopsychosocial model of interacting factors that contribute to functional outcomes in SSD: The importance of *Hippocampal Volume* suggests a contribution from biological factors influencing brain development and the possibility that there may be different neural signatures for different functional phenotypes. The importance of *Avolition* and *Anhedonia* suggests a contribution from psychological factors describing mental state. The importance of the *MSCEIT* and IRI items suggests the importance of social processing.

*Avolition* and *Anhedonia* loaded highly and were consistently selected in all of the analytical strategies, echoing the importance of negative symptoms for functioning in SSD, which has been demonstrated repeatedly^9,10,23–26,31^. Beyond their importance for functioning in general, our results further suggest that *Avolition* may play a more predominant role in *Role Functioning*, while *Anhedonia* plays an important role in *Independent* and *Social Functioning*. All three analytical strategies resulted in *Avolition* and *Anhedonia* loading in opposite directions when distinguishing *Cluster 2*, implying that they have opposite effects on the determination of Cluster 2 membership. This pattern is clarified by comparing *Avolition* and *Anhedonia* across the 3 clusters (Fig. 5). We found that *Avolition* was similar between *Cluster 1* (impaired phenotype) and *Cluster 2* (intermediate phenotype), but less severe in *Cluster 3* (resilient phenotype), matching the pattern we found for *Role Functioning*. Conversely, *Anhedonia* was similar between *Clusters 2* and *3*, but more impaired in *Cluster 1*, approximating the patterns for *Independent* and *Social Functioning*. The tight association between *Role Functioning* and *Avolition* has been noted previously^11,26^. It is also intuitive that motivation may play a key role in sustaining occupational and educational pursuits, while a better ability to experience and/or anticipate pleasure may feed into engagement in interpersonal and independent activities. The constructs of avolition and anhedonia can be interpreted to be overlapping with the idea of functional outcome. However, a key distinction is that negative symptoms primarily describe the internal state of the individual and, therefore, direct manifestations of schizophrenia, while functioning describes outwardly observable results and, therefore, should be considered outcomes. It may prove important to identify the critical areas of functioning in individual patients and selectively target the associated negative symptoms. These findings highlight the importance of ongoing investigations into psychosocial and pharmacological interventions for negative symptoms and suggest that distinctions among different areas of functioning and different domains of negative symptoms may be indicated when assessing the impact of these interventions on functional outcomes.

Of the other key correlates, the *MSCEIT* score was significantly higher in *Cluster 3* than in either of the other clusters. There is mounting evidence for strategies that target social cognitive and processing with benefits for functioning in SSD^56–58^. The remaining measures (*Left Hippocampal Volume, IRI Fantasy*, and *IRI Personal Distress)* did not show large group effects. Hippocampal volume reductions are among the most well-established anatomical findings in people with schizophrenia^59^ and have been associated with functioning, as well as psychosis severity^60–62^. Thus, it is unsurprising that hippocampal volume should emerge as a key predictor of functioning in this study. The lack of significant group effects for *Left Hippocampal Volume, IRI Fantasy*, and *IRI Personal Distress* most likely represent important higher-order interactions. The clinical significance of identifying higher-order interactions is that these may represent a means for identifying individuals who are most likely to benefit from intervention. For example, several psychosocial interventions have shown efficacy in improving negative symptoms in SSD^63^. The interactions present an interesting conjecture that functioning is more or less likely to be improved through negative symptom interventions depending on the individual’s hippocampal volume or baseline interpersonal attitudes. Performance on the *MSCEIT* was the only cognitive feature that appeared on both LDA approaches. However, *Processing Speed* and *Visual Learning* were selected by both the backward LDA and regularized regression approaches, and the regularized regressions also identified *Reasoning*, *WTAR Standard Score*, and *Verbal Learning* as potential predictors of functional phenotype. The finding that nonsocial cognitive features did not appear consistently on the LDAs may be explained by their covariance with negative symptoms and *MSCEIT* score (as many of them show group differences among the 3 clusters), as well as the proposition that the relationship between neurocognition and functioning is mediated by social cognition^46^.

Several important limitations should be considered. Both Datasets were evaluated at a single time point. Therefore, longitudinal studies are needed to evaluate whether the key correlates identified here are predictors or determinants of functioning in a prospective manner. Previous studies suggest this is the case for avolition and social cognition^9^. In both Datasets, functioning was determined primarily based on participant reports, which may lack objectivity. *Dataset II* was used to validate our findings for *Objective I* because it presented a convenient, available independent sample as there was overlap in the functioning constructs evaluated; however, some of the functioning items used in *Dataset II* may not be exactly equivalent or the best ways of measuring these constructs. In fact, we find it a strength of the findings that despite these inconsistencies in the ways that functioning was measured, we were able to identify consistent findings for *Objective I*. Our emphasis was on identifying reliable correlates of functional phenotype and not on constructing a predictive model to be used for prognostic purposes – this is an important, but distinct objective that should be independently pursued. We were not able to validate the findings from *Objective II* in an independent sample as we did for *Objective I* because we did not identify an additional dataset with the same range in biopsychosocial and functioning measures. Instead, reproducibility was emphasized with several layers of methodological cross-validation: identifying common findings from three separate analytical strategies, testing set-aside samples, and training the classification models using leave-one-out cross-validation. The 65 variables assessed in *Objective II* represent the most inclusive analysis of potential correlates of functioning in SSD to our knowledge, but to balance the breadth of variables explored with the resulting complexity of the findings, we did not consider some potentially important correlates. Brain-based variables were limited to volumetric measures because high-quality data was available for a greater number of participants and because these measures demonstrated more reproducible effects than structural and functional brain connectivity. Antipsychotic medication dosage and history were not reliably collected in the datasets. Because of the nature of the functioning assessments used, and the restriction of the study sites to North America, the functional phenotypes and correlates identified here may only apply to Western culture-bound standards of functioning^1^. A better understanding of other cultural contexts is required.

In summary, we define three functional phenotypes in schizophrenia-spectrum disorders, representing a relatively resilient phenotype, an impaired phenotype, and a previously under-recognized intermediate phenotype with impaired *Role Functioning* but partially preserved *Independent* and *Social Functioning*. Key correlates of functional phenotype span the biopsychosocial spectrum and prominently include *Avolition*, which appears to contribute most strongly to *Role Functioning*, and *Anhedonia*, which may play a large role in *Independent* and *Social Functioning*. Our findings support the continued development of interventions targeting negative symptoms due to their importance for functional outcomes and further suggest the possibility that different symptoms and functioning areas should be prioritized in different individuals.

### Supplementary information

Supplemental Materials

## Data Availability

The raw data for the SPINS study is available from the National Data Archive.
