# Supplementary material for: Functional phenotypes in schizophrenia spectrum disorders: defining the constructs and identifying biopsychosocial correlates using data-driven methods
Source: Schizophrenia (Heidelb). 2024 Jun 24;10(1):58. doi: 10.1038/s41537-024-00479-9 (PMC11196713; doi:10.1038/s41537-024-00479-9)
Supplement: Supplementary file 1 — Supplemental Materials [file 41537_2024_479_MOESM1_ESM.docx]

Functional Phenotypes in Schizophrenia Spectrum Disorders: Defining the Constructs and Identifying Biopsychosocial Correlates using Data-Driven Methods

*Sunny X. Tang, M.D.,* Katrin Hänsel, Ph.D.,* Lindsay Oliver, Ph.D., Erin Dickie, Ph.D., Colin Hawco, Ph.D., Majnu John, Ph.D., Aristotle Voineskos, M.D., Ph.D., Robert Buchanan, M.D., Anil K. Malhotra, M.D.*

** Drs. Tang and Hänsel contributed equally to the manuscript.*

Supplemental Materials

Methods

[Supplemental Methods – Functioning Assessments 2](#_Toc160571053)

[Supplemental Methods – Biopsychosocial Assessments 3](#_Toc160571054)

**Tables**

[Supplemental Table 1 – Objective I and II Methods: R Packages. 5](#_Toc160571638)

[Supplemental Table 2 – Objective II Methods: Sample Characteristics. 6](#_Toc160571639)

[Supplemental Table 3 – Objective II Methods: Binomial Coefficients for Backward-Elimination LDA. 7](#_Toc160571640)

[Supplemental Table 4 – Objective II Methods: Eliminated Variables for Backward-Elimination LDA. 9](#_Toc160571641)

[Supplemental Table 5 – Objective I Result: Functional Phenotype Clusters and Principal Components of Functioning. 11](#_Toc160571642)

[Supplemental Table 6 – Objective I Result: Functional Phenotype Clusters and Demographic and Clinical Characteristics. 13](#_Toc160571643)

[Supplemental Table 7 – Functional Cluster Group Effects for Biopsychosocial Predictors. 15](#_Toc160571644)

**Figures**

[Supplemental Figure 1 – Objective I Methods: PCA Pre-requisites and Scree Plot. 17](#_Toc160571674)

[Supplemental Figure 2 – Objective I Results: Overview of Functional Cluster Subitems. 18](#_Toc160571675)

[Supplemental References](#_Toc129863602) **17**

# Supplemental Methods – Functioning Assessments

In *Dataset I,* functioning was assessed with the Birchwood Social Functioning Scale (*BSFS*) (1) and Quality of Life Scale (*QoL*) (2). In *Dataset II,* related functioning domains were assessed: *work and interests* from Hamilton Rating Scale for Depression (*Ham-D*) (3), *Role* and *Residential Functioning* from Multidimensional Scale of Independent Functioning (*MSIF*) (4), *Leisure Activities*, *Social Frequency*, and *Degree of Social Activity* from Social Adjustment Scale (*SAS*) (5), and *Financial* and *Communication Skills* from Performance-Based Skills Assessment (*UPSA*) (4). Table 2 lists each functioning item. The i**ndividual items were used as the basis for both the clustering and PCA approaches in Objective I.**

The *BSFS*, *QoL*, *HAM-D*, and *MSIF* were administered by a trained research coordinator who asked standardized questions, prompted the participant as necessary for clarification, and completed ratings based on established criteria. The *BSFS* was designed to measure social skills and performance in schizophrenia; it has good psychometric properties (α=0.71-0.87; interrater reliability IRR=0.69-0.96) (1). The *QoL* was designed to measure functioning in the schizophrenia deficit syndrome; it has 4 latent factors represented by the subscales, and IRR=0.58-0.98 (6). The *HAM-D* is a highly-validated clinician-rated measure for depression, and includes one item on engagement in work and other activities (3,7). The *MSIF* was described to measure functional disability at work and at home in outpatients with psychiatric disorders; it has good psychometric properties (α=0.72, IRR=0.73-1.0) (8).

The *SAS* is a self-report instrument designed to measure social functioning in people with depression; each item is on a 5-point Likert scale, and the instrument has good psychometric properties (α=0.74, *r*=0.72 with clinical ratings) (5,9).

The *UPSA* is a performance-based skills assessment where participants are scored based on their ability to complete everyday tasks (or describe how they would be completed), e.g., making phone calls, preparing for a medical appointment, making change, paying a bill; the *UPSA* has good interrater reliability (IRR=0.91) (10).

# Supplemental Methods – Biopsychosocial Assessments

**The following assessments of biopsychosocial measures were performed for Objective II:**

- ***IRI* – Interpersonal Reactivity Index (11):** Self-reported ratings for subjective experience of interpersonal situations, such as perspective-taking - cognitive empathy (e.g., “putting yourself in others’ shoes”), fantasy – connecting with fictional or imagined experiences, empathic concern – emotional experience of empathy for others, personal distress – overwhelming emotional experiences during stressful situations.
- ***SPQ-B* – Schizotypal Personality Questionnaire-Brief (12):** Self-reported ratings for subjective experiences: cognitive-perceptual – subthreshold symptoms like altered sensorium, unusual, paranoid and referential thinking; interpersonal – discomfort with social situations and interpersonal relationships; disorganized – unusual behaviors and speech.
- ***BPRS* - Brief Psychiatric Rating Scale (13):** Semi-structured clinical interview with standardized ratings, completed by trained research coordinators. Overall’s four-factor model was used to represent individual symptom domains (14). The withdrawal/retardation factor was omitted to avoid duplication with the *SANS*.
- ***SANS* - Scale for the Assessment of Negative Symptoms (15):** Semi-structured clinical interview with standardized ratings, completed by trained research coordinators.
- ***MATRICS* - NIMH-Measurement and Treatment Research to Improve Cognition in Schizophrenia (16):** Objective multi-domain cognitive assessment, given and scored by a trained research coordinator.
- ***WTAR* - Wechsler Test of Adult Reading (17):** Word-list reading assessment given by a trained research coordinator, which assesses pre-morbid verbal IQ.
- ***MSCEIT* - Mayer-Salovey-Caruso Emotional Intelligence (18):** Objective assessment of emotional intelligence where the participant answers multiple-choice questions about a series of blurbs describing social and emotional situations.
- ***ER40* – Penn Emotion Recognition 40 Task (19):** Objective assessment of emotion processing where the participant identifies the emotion expressed in photographs displayed via a computer program. Accuracy and speed are measured separately.
- ***TASIT* - The Awareness of Social Inference Test (20):** Objective assessment of mental state attributions via multiple choice questions following videos depicting interpersonal exchanges.
- ***RMET* - Reading the Mind in the Eyes (21):** Objective assessment of mental state attributions; participants are presented with cropped depictions of eyes and are tasked to identify the corresponding emotional state.
- ***RAD* - Relationships Across Domains (22):** Objective assessment of social perception via multiple choice questions regarding verbal vignettes depicting dyadic interactions.
- ***MRI* – Magnetic Resonance Imaging:** Anatomical T1-weighted scans were collected using a fast-gradient sequence (sequence details below). Regional and global measures of brain volume were derived from T1-weighted images using the cortical Desikan-Killiany Atlas and the aseg subcortical parcellation in FreeSurfer 6.0.1 (23). MRI metrics from four participants were excluded due to poor T1-weighted image quality based on visual inspection.

| **T1 weighted Anatomical MRI** | | | | | | | | | | | | | | |
| --- | --- | --- | --- | --- | --- | --- | --- | --- | --- | --- | --- | --- | --- | --- |
| **Scanner ID** | **Plane** | **Imaging Mode** | **Phase Acquisition Order** | **Voxel Size (mm)** | **Matrix** | **FOV Read (mm)** | **Pulse Sequence** | **# Slices** | **TR (ms)** | **TE (ms)** | **TI (ms)** | **Flip Angle (deg)** | **Head Coil (channel)** |  |
| CMH | saggital | 3D | interleaved | 0.9 x 0.9 x 0.9 | 256 x 256 | 230 | BRAVO | 200 | 6.7 | 3 | 650 | 8 | 8 |  |
| MRC | saggital | 3D | interleaved | 0.9 x 0.9 x 0.9 | 256 x 256 | 230 | MPRAGE | 240 | 2300 | 2.9 | 900 | 9 | 12 |  |
| ZHH | saggital | 3D | interleaved | 0.9 x 0.9 x 0.9 | 256 x 256 | 230 | BRAVO | 200 | 6.4 | 2.8 | 650 | 8 | 8 |  |
| CMP | saggital | 3D | interleaved | 0.9 x 0.9 x 0.9 | 256 x 256 | 230 | MPRAGE | 240 | 2300 | 2.9 | 900 | 9 | 64 |  |
| MRP | saggital | 3D | interleaved | 0.9 x 0.9 x 0.9 | 256 x 256 | 230 | MPRAGE | 240 | 2300 | 2.9 | 900 | 9 | 64 |  |
| ZHP | saggital | 3D | interleaved | 0.9 x 0.9 x 0.9 | 256 x 256 | 230 | MPRAGE | 240 | 2300 | 2.9 | 900 | 9 | 64 |  |
| * GE BRAVO sequences report TR as the time between small flip angle pulses, rather than the time between inversion pulses | | | | | | | | | | | | | | |

Supplemental Table 1 – Objective I and II Methods: R Packages.

The following R packages were used for analysis in *Objectives I* and *II* as follows:

| Package | Application | Version | Reference | Objective |
| --- | --- | --- | --- | --- |
| NbClust | NbClust implements 30 metrics to determine an optimal cluster schema. | 3.0 | (24) | I |
| stats | Hierarchical Clustering using the Ward method. R base package. | 4.1.2 |  | I |
| fpc | Bootstrapped hierarchical clustering and assessment of cluster stability. | 2.2-12 | (25) | I |
| psych | Principal Component Analysis, including Bartlett’s test of sphericity and Kaiser-Meyer-Olkin measures | 2.2.3 | (26) | I |
| mice | Multiple imputation of missing values | 3.14.0 | (27) | II |
| MASS | Linear Discriminant Analysis (LDA) | 7.3.58.1 | (28) | II |
| glmnet | Regularized regressions in form of LASSO and RIDGE regression | 4.1.3 | (29) | II |
| rstatix | Basic statistical tests | 0.7.0 | (30) | I & II |

Supplemental Table 2 – Objective II Methods: Sample Characteristics.

The table shows the demographic characteristics of the train and test set, as well as, the functional phenotypes: Cluster 1 – impaired phenotype across the social, independent, and role functional domains; Cluster 2 – intermediate phenotype with impaired role functioning but partially preserved social and independent functioning; Cluster 3 – resilient phenotype with higher role, independent and social functioning.

|  |  | Training Set  (*n* = 199, 80%) |  | Test Set (*n* = 49, 20%) |  | p |  | Cohen’s *D* |
| --- | --- | --- | --- | --- | --- | --- | --- | --- |
| Functional Phenotype – n (%) |  |  |  |  |  | 0.67 |  |  |
| Cluster 1 |  | 53 (26.6%) |  | 11 (22.4.0%) |  |  |  |  |
| Cluster 2 |  | 68 (34.2%) |  | 20 (40.8%) |  |  |  |  |
| Cluster 3 |  | 78 (39.2%) |  | 18 (36.7%) |  |  |  |  |
| Age – Years (SD) |  | 32.4 (10.4) |  | 30.4 (9.0) |  | 0.21 |  | 0.20 |
| Sex – n Female (%) |  | 70 (35.2%) |  | 11 (22.4%) |  | 0.09 |  |  |
| Race – n (%) |  |  |  |  |  | 0.66 |  |  |
| Asian |  | 29 (14.6%) |  | 6 (12.2%) |  |  |  |  |
| Black |  | 57 (28.6%) |  | 17 (32.7%) |  |  |  |  |
| White |  | 101 (50.8%) |  | 26 (53.1%) |  |  |  |  |
| Other |  | 12 (6.0%) |  | 1 (2.0%) |  |  |  |  |
| Hispanic Ethnicity -– n (%) |  | 23 (11.6%) |  | 4 (8.2%) |  | 0.49 |  |  |
| Diagnosis – n (%) |  |  |  |  |  | 0.05 |  |  |
| Schizophrenia |  | 138 (69.3%) |  | 45 (91.8%) |  |  |  |  |
| Schizoaffective Disorder |  | 44 (22.1%) |  | 1 (2.0%) |  |  |  |  |
| Schizophreniform Disorder |  | 6 (3 %) |  | 0 (0.0%) |  |  |  |  |
| Psychosis NOS |  | 11 (5.5%) |  | 3 (6.1%) |  |  |  |  |
| *BPRS* Total Score (SD) |  | 30.80 (7.49) |  | 32.59 (8.87) |  | 0.15 |  | -0.23 |
| *SANS* Global Total (SD) |  | 7.76 (3.44) |  | 7.53 (2.78) |  | 0.63 |  | -0.07 |

Supplemental Table 3 – Objective II Methods: Binomial Coefficients for Backward-Elimination LDA.

An overview of binomial coefficients $\left( \begin{matrix} n \\ k \end{matrix} \right)$ ($n$ over $k$) that signify the number of combinations that need to be considered when choosing $k$ variables out of $n$. Color highlighted with orange shading are the combinations that exceed our computational threshold of 2M combinations.

| n \ k | 1 | 2 | 3 | 4 | 5 | 6 | 7 | 8 | 9 | 10 |
| --- | --- | --- | --- | --- | --- | --- | --- | --- | --- | --- |
| 65 | 65 | 2,080 | 43,680 | 677,040 | 8,259,888 | 82,598,880 | 696,190,560 | 5,047,381,560 | 31,966,749,880 | 179,013,799,328 |
| 64 | 64 | 2,016 | 41,664 | 635,376 | 7,624,512 | 74,974,368 | 621,216,192 | 4,426,165,368 | 27,540,584,512 | 151,473,214,816 |
| 63 | 63 | 1,953 | 39,711 | 595,665 | 7,028,847 | 67,945,521 | 553,270,671 | 3,872,894,697 | 23,667,689,815 | 127,805,525,001 |
| 62 | 62 | 1,891 | 37,820 | 557,845 | 6,471,002 | 61,474,519 | 491,796,152 | 3,381,098,545 | 20,286,591,270 | 107,518,933,731 |
| 61 | 61 | 1,830 | 35,990 | 521,855 | 5,949,147 | 55,525,372 | 436,270,780 | 2,944,827,765 | 17,341,763,505 | 90,177,170,226 |
| 60 | 60 | 1,770 | 34,220 | 487,635 | 5,461,512 | 50,063,860 | 386,206,920 | 2,558,620,845 | 14,783,142,660 | 75,394,027,566 |
| 59 | 59 | 1,711 | 32,509 | 455,126 | 5,006,386 | 45,057,474 | 341,149,446 | 2,217,471,399 | 12,565,671,261 | 62,828,356,305 |
| 58 | 58 | 1,653 | 30,856 | 424,270 | 4,582,116 | 40,475,358 | 300,674,088 | 1,916,797,311 | 10,648,873,950 | 52,179,482,355 |
| 57 | 57 | 1,596 | 29,260 | 395,010 | 4,187,106 | 36,288,252 | 264,385,836 | 1,652,411,475 | 8,996,462,475 | 43,183,019,880 |
| 56 | 56 | 1,540 | 27,720 | 367,290 | 3,819,816 | 32,468,436 | 231,917,400 | 1,420,494,075 | 7,575,968,400 | 35,607,051,480 |
| 55 | 55 | 1,485 | 26,235 | 341,055 | 3,478,761 | 28,989,675 | 202,927,725 | 1,217,566,350 | 6,358,402,050 | 29,248,649,430 |
| 54 | 54 | 1,431 | 24,804 | 316,251 | 3,162,510 | 25,827,165 | 177,100,560 | 1,040,465,790 | 5,317,936,260 | 23,930,713,170 |
| 53 | 53 | 1,378 | 23,426 | 292,825 | 2,869,685 | 22,957,480 | 154,143,080 | 886,322,710 | 4,431,613,550 | 19,499,099,620 |
| 52 | 52 | 1,326 | 22,100 | 270,725 | 2,598,960 | 20,358,520 | 133,784,560 | 752,538,150 | 3,679,075,400 | 15,820,024,220 |
| 51 | 51 | 1,275 | 20,825 | 249,900 | 2,349,060 | 18,009,460 | 115,775,100 | 636,763,050 | 3,042,312,350 | 12,777,711,870 |
| 50 | 50 | 1,225 | 19,600 | 230,300 | 2,118,760 | 15,890,700 | 99,884,400 | 536,878,650 | 2,505,433,700 | 10,272,278,170 |
| 49 | 49 | 1,176 | 18,424 | 211,876 | 1,906,884 | 13,983,816 | 85,900,584 | 450,978,066 | 2,054,455,634 | 8,217,822,536 |
| 48 | 48 | 1,128 | 17,296 | 194,580 | 1,712,304 | 12,271,512 | 73,629,072 | 377,348,994 | 1,677,106,640 | 6,540,715,896 |
| 47 | 47 | 1,081 | 16,215 | 178,365 | 1,533,939 | 10,737,573 | 62,891,499 | 314,457,495 | 1,362,649,145 | 5,178,066,751 |
| 46 | 46 | 1,035 | 15,180 | 163,185 | 1,370,754 | 9,366,819 | 53,524,680 | 260,932,815 | 1,101,716,330 | 4,076,350,421 |
| 45 | 45 | 990 | 14,190 | 148,995 | 1,221,759 | 8,145,060 | 45,379,620 | 215,553,195 | 886,163,135 | 3,190,187,286 |
| 44 | 44 | 946 | 13,244 | 135,751 | 1,086,008 | 7,059,052 | 38,320,568 | 177,232,627 | 708,930,508 | 2,481,256,778 |
| 43 | 43 | 903 | 12,341 | 123,410 | 962,598 | 6,096,454 | 32,224,114 | 145,008,513 | 563,921,995 | 1,917,334,783 |
| 42 | 42 | 861 | 11,480 | 111,930 | 850,668 | 5,245,786 | 26,978,328 | 118,030,185 | 445,891,810 | 1,471,442,973 |
| 41 | 41 | 820 | 10,660 | 101,270 | 749,398 | 4,496,388 | 22,481,940 | 95,548,245 | 350,343,565 | 1,121,099,408 |
| 40 | 40 | 780 | 9,880 | 91,390 | 658,008 | 3,838,380 | 18,643,560 | 76,904,685 | 273,438,880 | 847,660,528 |
| 39 | 39 | 741 | 9,139 | 82,251 | 575,757 | 3,262,623 | 15,380,937 | 61,523,748 | 211,915,132 | 635,745,396 |
| 38 | 38 | 703 | 8,436 | 73,815 | 501,942 | 2,760,681 | 12,620,256 | 48,903,492 | 163,011,640 | 472,733,756 |
| 37 | 37 | 666 | 7,770 | 66,045 | 435,897 | 2,324,784 | 10,295,472 | 38,608,020 | 124,403,620 | 348,330,136 |
| 36 | 36 | 630 | 7,140 | 58,905 | 376,992 | 1,947,792 | 8,347,680 | 30,260,340 | 94,143,280 | 254,186,856 |
| 35 | 35 | 595 | 6,545 | 52,360 | 324,632 | 1,623,160 | 6,724,520 | 23,535,820 | 70,607,460 | 183,579,396 |
| 34 | 34 | 561 | 5,984 | 46,376 | 278,256 | 1,344,904 | 5,379,616 | 18,156,204 | 52,451,256 | 131,128,140 |
| 33 | 33 | 528 | 5,456 | 40,920 | 237,336 | 1,107,568 | 4,272,048 | 13,884,156 | 38,567,100 | 92,561,040 |
| 32 | 32 | 496 | 4,960 | 35,960 | 201,376 | 906,192 | 3,365,856 | 10,518,300 | 28,048,800 | 64,512,240 |
| 31 | 31 | 465 | 4,495 | 31,465 | 169,911 | 736,281 | 2,629,575 | 7,888,725 | 20,160,075 | 44,352,165 |
| 30 | 30 | 435 | 4,060 | 27,405 | 142,506 | 593,775 | 2,035,800 | 5,852,925 | 14,307,150 | 30,045,015 |
| 29 | 29 | 406 | 3,654 | 23,751 | 118,755 | 475,020 | 1,560,780 | 4,292,145 | 10,015,005 | 20,030,010 |
| 28 | 28 | 378 | 3,276 | 20,475 | 98,280 | 376,740 | 1,184,040 | 3,108,105 | 6,906,900 | 13,123,110 |
| 27 | 27 | 351 | 2,925 | 17,550 | 80,730 | 296,010 | 888,030 | 2,220,075 | 4,686,825 | 8,436,285 |
| 26 | 26 | 325 | 2,600 | 14,950 | 65,780 | 230,230 | 657,800 | 1,562,275 | 3,124,550 | 5,311,735 |
| 25 | 25 | 300 | 2,300 | 12,650 | 53,130 | 177,100 | 480,700 | 1,081,575 | 2,042,975 | 3,268,760 |
| 24 | 24 | 267 | 2,024 | 10,626 | 42,504 | 134,596 | 346,104 | 735,471 | 1,307,504 | 1,961,256 |

Supplemental Table 4 – Objective II Methods: Eliminated Variables for Backward-Elimination LDA.

**For each level of the backward-selection LDA, the number of original predictors** $n$**, selected predictors** $k$ **(which increment per level), and number of predictor combinations are shown. In case that the number of combinations exceed the upper boundary of 2M, we reduce the number of predictors until the criteria is met. E.g., level 5 would mean a selection of 5 variables out of 65 predictors resulting in over 8M combinations to test. We eliminate 16 predictors to reduce the number of combinations below 2M (5 out of 49).**

| Level | eliminated variables | $n$  (total predictors) | $k$  (selected predictors) | $\left( \begin{matrix} n \\ k \end{matrix} \right)$  (number of combinations) |
| --- | --- | --- | --- | --- |
| 1 | - | 65 | 1 | 65 |
| 2 | - | 65 | 2 | 2,080 |
| 3 | - | 65 | 3 | 43,680 |
| 4 | - | 65 | 4 | 677,040 |
| 5 | SYMP: BPRS Anx. and Dep.  DEMO: Age  DEMO: Asian Race  DEMO: Sex  DEMO: Duratin of Illness  SCOG: ER40 Accuracy  SCOG: ER40 Speed  MRI: Total Brain Vol.  MRI: L Thalamic Vol.  MRI: L Lateral Orbitofrontal Vol.  MRI: L Superior Frontal Vol.  MRI: Prefrontal Cortices Vol.  MRI: R Inferior Frontal Vol.  MRI: R Medial Orbitofrontal Vol.  MRI: R Rostral Middle Frontal Vol.  SYMP: SANS Aff. Flattening | 49 | 5 | 1,906,884 |
| 6 | SYMP: BPRS Pos. Symptoms  DEMO: Parental Education  DEMO: Hispanic  DEMO: White Race  MRI: L Amygdala Vol.  MRI: R Entorhinal Vol.  MRI: L Medial Orbitofrontal Vol.  MRI: L Rostral Middle Frontal Vol.  MRI: R Caudal Middle Frontal Vol.  MRI: Total Gray Matter Vol.  SELF: IRI Empathic Concern  SCOG: TASIT  COG: WTAR Std. Score | 36 | 6 | 1,947,792 |
| 7 | MRI: R Lateral Orbitofrontal Vol.  DEMO: Black Race  MRI: L Caudal Middle Frontal Vol.  MRI: L Inferior Frontal Vol.  MRI: R Superior Frontal Vol.  MRI: R Thalamic Vol.  COG: Working Memory | 29 | 7 | 1,560,780 |
| 8 | MRI: Total Ventricular Vol.  COG: Reasoning  SCOG: RAD Total Score | 26 | 8 | 1,562,275 |
| 9 | SYMP: BPRS Activation  MRI: L Entorhinal Vol. | 24 | 9 | 1,307,504 |
| 10 | - | 24 | 10 | 1,961,256 |

Supplemental Table 5 – Objective I Result: Functional Phenotype Clusters and Principal Components of Functioning.

Sub-tables A (*Dataset I*) and B (*Dataset II*) show the descriptive statistics of the 3 functional components, i.e., *Independent*, *Social*, and *Role Functioning*, across the three outcome clusters. After visually inspecting the data for normality, pairwise t-tests between the 3 clusters were performed after adjusting $\alpha$ using the Bonferroni-Holm method (31) (n.s. – not significant; * $p<0.05$; ** $p<0.01$; *** $p<0.001$). Note: *Cluster 1* – impaired phenotype across the social, independent, and role functional domains; *Cluster 2* – intermediate phenotype with impaired role functioning but partially preserved social and independent functioning; *Cluster 3* – resilient phenotype with higher role, independent and social functioning.

| A - Dataset I | | | | | | | | | | | | | | | | | |
| --- | --- | --- | --- | --- | --- | --- | --- | --- | --- | --- | --- | --- | --- | --- | --- | --- | --- |
|  |  | Cluster 1 (n = 71, 25%) | |  | Cluster 2 (n=102, 36%) | |  | Cluster 3 (n=109, 39%) | |  | Group Comparisons | | | | | |  |
|  |  | mean | SD |  | mean | SD |  | mean | SD |  | t | p | Signf. | CI | Cohen's *D* | magnitude | |
| Independent Functioning | | -0.97 | 0.76 |  | -0.09 | 0.73 |  | 0.71 | 0.77 |  |  | | | | | | |
|  | 1 vs 2 |  |  |  |  |  |  |  |  |  | -7.7 | <0.001 | *** | [-1.53,-0.89] | -1.20 | large | |
|  | 2 vs 3 |  |  |  |  |  |  |  |  |  | -7.8 | <0.001 | *** | [-1.34,-0.81] | -1.07 | large | |
|  | 1 vs 3 |  |  |  |  |  |  |  |  |  | -14.5 | <0.001 | *** | [-2.57,-1.89] | -2.21 | large | |
| Social Functioning | | -1.18 | 0.80 |  | 0.2 | 0.60 |  | 0.58 | 0.75 |  |  | | | | | | |
|  | 1 vs 2 |  |  |  |  |  |  |  |  |  | -12.4 | <0.001 | *** | [-2.35,-1.64] | -1.96 | large | |
|  | 2 vs 3 |  |  |  |  |  |  |  |  |  | -4.1 | 0.001 | *** | [-0.9,-0.28] | -0.56 | moderate | |
|  | 1 vs 3 |  |  |  |  |  |  |  |  |  | -14.8 | <0.001 | *** | [-2.7,-1.93] | -2.27 | large | |
| Role Functioning | | -0.72 | 0.75 |  | -0.56 | 0.55 |  | 1.00 | 0.55 |  |  | | | | | | |
|  | 1 vs 2 |  |  |  |  |  |  |  |  |  | -1.5 | 0.14 | n.s. | [-0.58,0.05] | -0.23 | small | |
|  | 2 vs 3 |  |  |  |  |  |  |  |  |  | -20.6 | <0.001 | *** | [-3.26,-2.47] | -2.83 | large | |
|  | 1 vs 3 |  |  |  |  |  |  |  |  |  | -16.7 | <0.001 | *** | [-3.18,-2.23] | -2.62 | large | |

| B - Dataset II | | | | | | | | | | | | | | | | | | | |
| --- | --- | --- | --- | --- | --- | --- | --- | --- | --- | --- | --- | --- | --- | --- | --- | --- | --- | --- | --- |
|  |  | Cluster 1  (n = 104, 33%) | |  | Cluster 2  (n=184, 58%) | |  | Cluster 3 (n=29, 9%) | | |  | Group Comparisons | | | | | | |  |
|  |  | mean | SD |  | mean | SD |  | mean | SD | |  | t | p | Signf. | CI | Cohen's *D* | magnitude | | |
| Independent Functioning | | -0.61 | 1.02 |  | 0.12 | 0.72 |  | 1.40 | 0.72 |  |  | | | | | | |  |  |
|  | 1 vs 2 |  |  |  |  |  |  |  |  | |  | -6.5 | <0.001 | *** | [-1.10,-0.60] | -0.83 | large | | |
|  | 2 vs 3 |  |  |  |  |  |  |  |  | |  | -8.9 | <0.001 | *** | [-2.38,-1.36] | -1.78 | large | | |
|  | 1 vs 3 |  |  |  |  |  |  |  |  | |  | -12.1 | <0.001 | *** | [-2.78,-1.90] | -2.29 | large | | |
| Social Functioning | | -0.86 | 0.84 |  | 0.36 | 0.75 |  | 0.83 | 0.87 |  |  | | | | | | |  |  |
|  | 1 vs 2 |  |  |  |  |  |  |  |  | |  | -12.3 | <0.001 | *** | [-1.84,-1.28] | -1.54 | large | | |
|  | 2 vs 3 |  |  |  |  |  |  |  |  | |  | -2.8 | 0.01 | ** | [-1.07,-0.17] | -0.59 | moderate | | |
|  | 1 vs 3 |  |  |  |  |  |  |  |  | |  | -9.4 | <0.001 | *** | [-2.64,-1.49] | -1.98 | large | | |
| Role Functioning | | -0.08 | 0.58 |  | -0.19 | 1.02 |  | 1.50 | 0.82 |  |  | | | | | | |  |  |
|  | 1 vs 2 |  |  |  |  |  |  |  |  | |  | 1.2 | 0.23 | n.s. | [-0.09,0.37] | 0.14 | negligible | | |
|  | 2 vs 3 |  |  |  |  |  |  |  |  | |  | -9.9 | <0.001 | *** | [-2.31,-1.40] | -1.83 | large | | |
|  | 1 vs 3 |  |  |  |  |  |  |  |  | |  | -9.7 | <0.001 | *** | [-3.17,-1.60] | -2.21 | large | | |

Supplemental Table 6 – Objective I Result: Functional Phenotype Clusters and Demographic and Clinical Characteristics.

Sub-Tables A (*Dataset I*) and B (*Dataset II*) present demographic and clinical characteristics for the 3 phenotype clusters. Continuous variables were evaluated with ANOVA and categorical variables were evaluated with Fisher’s Exact Test.

A - Dataset I

| Variable |  | Cluster 1 (n = 71, 25%) |  | Cluster 2 (n=102, 36%) |  | Cluster 3 (n=109, 39%) |  | *p* |
| --- | --- | --- | --- | --- | --- | --- | --- | --- |
| Age - Years (SD) |  | 31.78 (10.3) |  | 33.73 (10.1) |  | 31.0 (10.2) |  | 0.15 |
| Sex - n Female (%) |  | 19 (26.8%) |  | 42 (41.2%) |  | 36 (33.0%) |  | 0.13 |
| Race |  |  |  |  |  |  |  | 0.08 |
| White |  | 27 (38.0%) |  | 54 (52.9%) |  | 63 (57.8%) |  |  |
| Black |  | 27 (38.0%) |  | 29 (28.4%) |  | 30 (27.5%) |  |  |
| Asian |  | 13 (18.3%) |  | 10 (9.8%) |  | 13 (11.9%) |  |  |
| Other |  | 4 (5.6%) |  | 9 (8.8%) |  | 3 (2.8%) |  |  |
| Diagnosis - n (%) |  |  |  |  |  |  |  | 0.39 |
| Schizophrenia |  | 54 (76.1%) |  | 77 (75.5%) |  | 75 (68.8%) |  |  |
| Schizoaffective Disorder |  | 12 (16.9%) |  | 20 (19.6%) |  | 22 (20.2%) |  |  |
| Schizophreniform Disorder |  | 3 (4.2%) |  | 2 (2.0%) |  | 2 (1.8%) |  |  |
| Psychosis NOS |  | 2 (2.8%) |  | 3 (2.9%) |  | 10 (9.2%) |  |  |
| BPRS Total Score (SD) |  | 35.3 (8.2) |  | 30.6 (7.0) |  | 28.3 (6.8) |  | <0.001 |
| SANS Global Total (SD) |  | 10.4 (2.9) |  | 8.0 (2.6) |  | 5.5 (2.5) |  | <0.001 |
| *Note*: There were individuals with missing BPRS Total Scores (1 individual in Cluster 3) and SANS Global Total (2 individuals in Cluster 3). | | | | | | | | |

B - Dataset II

| Variable |  | Cluster 1 (n = 104, 33%) |  | Cluster 2 (n=184, 58%) |  | Cluster 3 (n=29, 9%) |  | *p* |
| --- | --- | --- | --- | --- | --- | --- | --- | --- |
| Age - Years (SD) |  | 43.1 (10.1) |  | 43.8 (10.1) |  | 41.0 (10.2) |  | 0.28 |
| Sex - n Female (%) |  | 22 (21.2%) |  | 59 (32.1%) |  | 6 (20.7%) |  | 0.11 |
| Race |  |  |  |  |  |  |  | 0.003 |
| White |  | 43 (41.3%) |  | 103 (56.0%) |  | 18 (62.1%) |  | 0.03 |
| Black |  | 57 (54.8%) |  | 63 (34.2%) |  | 10 (34.5%) |  | 0.002 |
| Other |  | 4 (3.8%) |  | 17 (9.2%) |  | 0 (0.0%) |  | 0.08 |
| Unknown |  | 0 (0.0%) |  | 1 (0.5%) |  | 1 (3.4%) |  | 0.17 |
| Diagnosis - n (%) |  |  |  |  |  |  |  | 0.15 |
| Schizophrenia |  | 94 (90.4%) |  | 153 (83.2%) |  | 23 (79.3%) |  |  |
| Schizoaffective Disorder |  | 10 (9.6%) |  | 31 (16.8%) |  | 6 (20.7%) |  |  |
| BPRS Total Score (SD) |  | 32.3 (7.8) |  | 30.8 (8.1) |  | 29.6 (6.6) |  | 0.17 |
| SANS Global Total (SD) |  | 9.4 (2.6) |  | 8.1 (3.1) |  | 6.5 (2.7) |  | <0.001 |
| *Note*: There were individuals with missing BPRS Total Scores (4 individuals in Cluster 1, 6 in Cluster 2, 1 in Cluster 3). | | | | | | | | |

Supplemental Table 7 – Functional Cluster Group Effects for Biopsychosocial Predictors.

The 65 biopsychosocial predictors from *Objective II* were evaluated. Presented below are biopsychosocial predictors with significant group effects (p < 0.05) for Functional Cluster. No correction for multiple comparisons was applied.

|  | **Cluster 1** | **Cluster 2** | **Cluster 3** |  |
| --- | --- | --- | --- | --- |
|  | (n = 64, 26%) | (n=88, 36%) | (n=96, 38%) | p |
| **DEMO: Sociodemographic & Personal Characteristics** | | | | |
| Race |  |  |  |  |
| White (%) | 25 (39.1%) | 45 (51.1%) | 57 (59.4%) | 0.042 |
| **COG: General Neurocognition (MATRICS)** | | | | |
| WTAR Std. Score (SD, n miss) | 104.3 (14.0, 1) | 102.7 (15.3, 0) | 110.5 (13.2, 0) | < 0.001 |
| Processing Speed – (SD) | 37.7 (13.9) | 36.5 (11.8) | 43.7 (12.2) | < 0.001 |
| Attention (SD, n miss) | 38.2 (13.5, 3) | 37.5 (11.3, 1) | 42.2 (10.3, 1) | 0.015 |
| Working Memory (SD) | 40.0 (10.5) | 39.2 (10.6) | 44.5 (11.2) | 0.002 |
| Verbal Learning (SD) | 38.7 (8.7) | 38.5 (7.7) | 43.0 (9.4) | < 0.001 |
| Visual Learning (SD) | 36.4 (12.7) | 36.9 (11.5) | 42.6 (11.9) | < 0.001 |
| Reasoning (SD) | 40.1 (11.4) | 40.6 (10.1) | 46.8 (10.0) | < 0.001 |
| **SCOG: Social cognition** | | | | |
| MSCEIT (SD, n miss) | 40.3 (13.5, 2) | 41.7 (11.1, 0) | 48.3 (10.4, 1) | < 0.001 |
| ER40 Speed (SD, n miss) | 2609.9 (866.1, 1) | 2415.9 (752.3, 1) | 2270.7 (718.5, 1) | 0.027 |
| TASIT (SD, n miss) | 113.7 (18.9, 2) | 115.9 (16.6, 0) | 122.9 (16.0, 1) | 0.002 |
| RMET (SD) | 24.0 (4.7) | 23.3 (5.8) | 25.8 (4.8) | 0.003 |
| RAD Total Score (SD, n miss) | 51.1 (10.0, 3) | 50.1 (9.1, 0) | 54.4 (8.5, 1) | 0.005 |
| **SELF: Subjective Psychological Experiences** | | | | |
| IRI Perspective Taking (SD) | 17.1 (4.9) | 19.3 (4.0) | 19.5 (4.7) | 0.002 |
| IRI Fantasy (SD) | 14.3 (6.2) | 15.7 (6.2) | 17.6 (5.6) | 0.003 |
| IRI Empathic Concern (SD) | 18.7 (4.7) | 20.8 (5.0) | 20.8 (4.8) | 0.012 |
| **SYMP: Clinical Ratings of Psychosis Symptoms** | | | | |
| BPRS Anx. and Dep. (SD) | 8.6 (3.8) | 7.6 (3.5) | 6.9 (2.7) | 0.009 |
| BPRS Pos. Symptoms (SD) | 9.4 (4.3) | 7.7 (3.6) | 7.0 (3.6) | < 0.001 |
| BPRS Hostility (SD) | 5.5 (2.3) | 4.7 (1.7) | 4.6 (1.8) | 0.017 |
| SANS Aff. Flattening (SD) | 2.4 (1.2) | 2.1 (1.1) | 1.5 (1.1) | < 0.001 |
| SANS Alogia (SD) | 1.5 (1.2) | 0.8 (0.9) | 0.6 (0.9) | < 0.001 |
| SANS Avolition (SD) | 3.5 (1.0) | 3.0 (1.0) | 1.7 (1.2) | < 0.001 |
| SANS Anhedonia (SD) | 3.2 (0.8, 0) | 2.1 (0.9, 0) | 1.7 (1.1, 1) | < 0.001 |
| SPQ Interpersonal (SD) | 5.0 (2.1, 1) | 4.1 (2.3, 1) | 3.5 (2.4, 1) | < 0.001 |
| SPQ Disorganized (SD) | 2.8 (2.0, 1) | 1.8 (1.9, 1) | 2.1 (1.8, 0) | 0.009 |
| **MRI: Structural Brain Imaging** | | | | |
| Total Brain Vol. – cc (SD) | 1142.9 (109.8) | 1130.6 (118.7) | 1188.2 (116.1) | 0.002 |
| Total Gray Matter Vol. – cc (SD) | 653.5 (68.3) | 641.315.6 (70.631.3) | 679.272.5 (69.157.9) | < 0.001 |
| R Superior Temporal Vol. – cc (SD) | 12.1 (1.8) | 11.9 (1.6) | 12.6 (1.7) | 0.031 |
| L Superior Temporal Vol. – cc (SD) | 13.2 (2.0) | 12.9 (1.8) | 13.6 (1.5) | 0.016 |
| R Entorhinal Vol. – cc (SD) | 1.8 (0.4) | 1.8 (0.5) | 2.0 (0.6) | 0.033 |
| L Hippocampal Vol. – cc (SD) | 3.7 (0.4) | 3.8 (0.5) | 3.9 (0.4) | 0.008 |
| R Hippocampal Vol. – cc (SD) | 3.9 (0.5) | 4.0 (0.4) | 4.1 (0.4) | 0.008 |
| R Amygdala Vol. – cc (SD) | 1.6 (0.2) | 1.6 (0.2) | 1.7 (0.2) | 0.003 |
| L Thalamic Vol. – cc (SD) | 7.6 (0.8) | 7.6 (0.9) | 8.0 (0.8) | 0.002 |
| R Thalamic Vol. – cc (SD) | 7.2 (0.8) | 7.1 (0.9) | 7.5 (0.8) | 0.026 |
| L Superior Frontal Vol. – cc (SD) | 23.2 (3.7) | 22.8 (3.4) | 24.5 (3.4) | 0.003 |
| R Superior Frontal Vol. – cc (SD) | 21.8 (3.5) | 21.6 (3.4) | 23.4 (3.5) | < 0.001 |
| R Caudal Middle Frontal Vol. – cc (SD) | 6.2 (1.3) | 6.0 (1.3) | 6.4 (1.2) | 0.099 |
| R Lateral Orbitofrontal Vol. – cc (SD) | 7.8 (1.4) | 7.8 (1.3) | 8.3 (1.2) | 0.012 |
| R Medial Orbitofrontal Vol. – cc (SD) | 5.6 (0.8) | 5.5 (0.8) | 5.9 (0.9) | 0.015 |
| R Rostral Middle Frontal Vol. – cc (SD) | 16.7 (3.1) | 16.5 (2.9) | 17.6 (3.2) | 0.039 |
| L Inferior Frontal Vol. – cc (SD) | 11.2 (1.7) | 11.0 (1.7) | 11.7 (1.8) | 0.024 |
| R Inferior Frontal Vol. – cc (SD) | 11.4 (1.8) | 11.1 (1.6) | 12.0 (1.9) | 0.003 |
| Prefrontal Cortices Vol. – cc (SD) | 70.3 (10.2) | 69.2 (9.8) | 73.8 (9.7) | 0.005 |
| Note: MATRICS domain scores are reported as standardized t-scores. Structural Brain Imaging measures are reported in cubic centimeter (cc). | | | | |

Supplemental Figure 1 – Objective I Methods: PCA Pre-requisites and Scree Plot.

A

B

Subfigures A.I and A.II show the pre-required test indicating the suitability to perform a PCA. *Datasets I* passed the tests, namely Bartlett’s Test of Sphericity ($p<0.05$), Kaiser-Meyer-Olkin Test (KMO; value>0.08), and Determinant of the correlation matrix is above 0.00001. *Dataset II* passed all tests apart the KMO test. Subplots B.I and B.II show the Scree plot of the Eigenvalues per component. The Kaiser’s rule, i.e., Eigenvalue>1, has been applied to identify the optimal number of components. The optimal number of components has been determined as 3 components for both datasets.


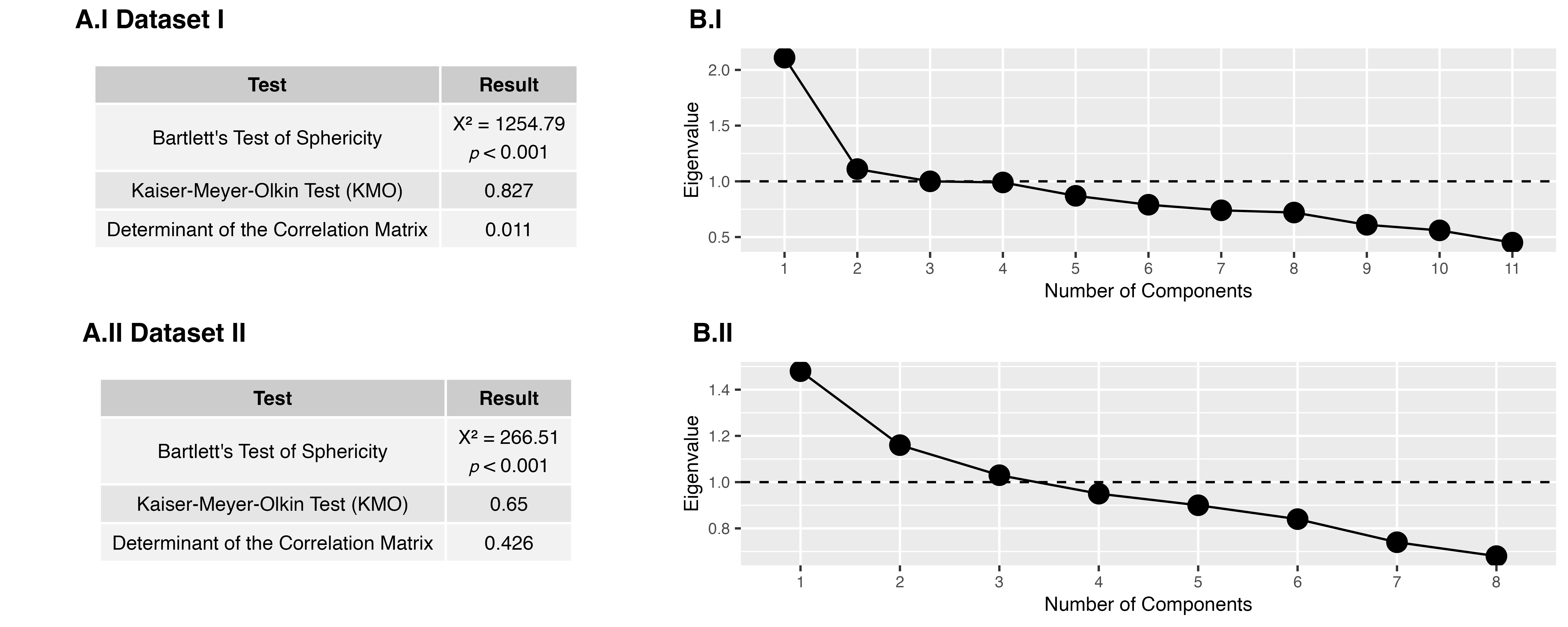


Supplemental Figure 2 – Objective I Results: Overview of Functional Cluster Subitems.

Dataset I

Dataset II

The following plot shows the standardized functional outcomes sub-item scores per functioning cluster. Z-score has been used as a standardization approach. Note: The functional phenotype clusters are: *Cluster 1* – impaired phenotype across the social, independent, and role functional domains; *Cluster 2* – intermediate phenotype with impaired role functioning but partially preserved social and independent functioning; *Cluster 3* – resilient phenotype with higher role, independent and social functioning.


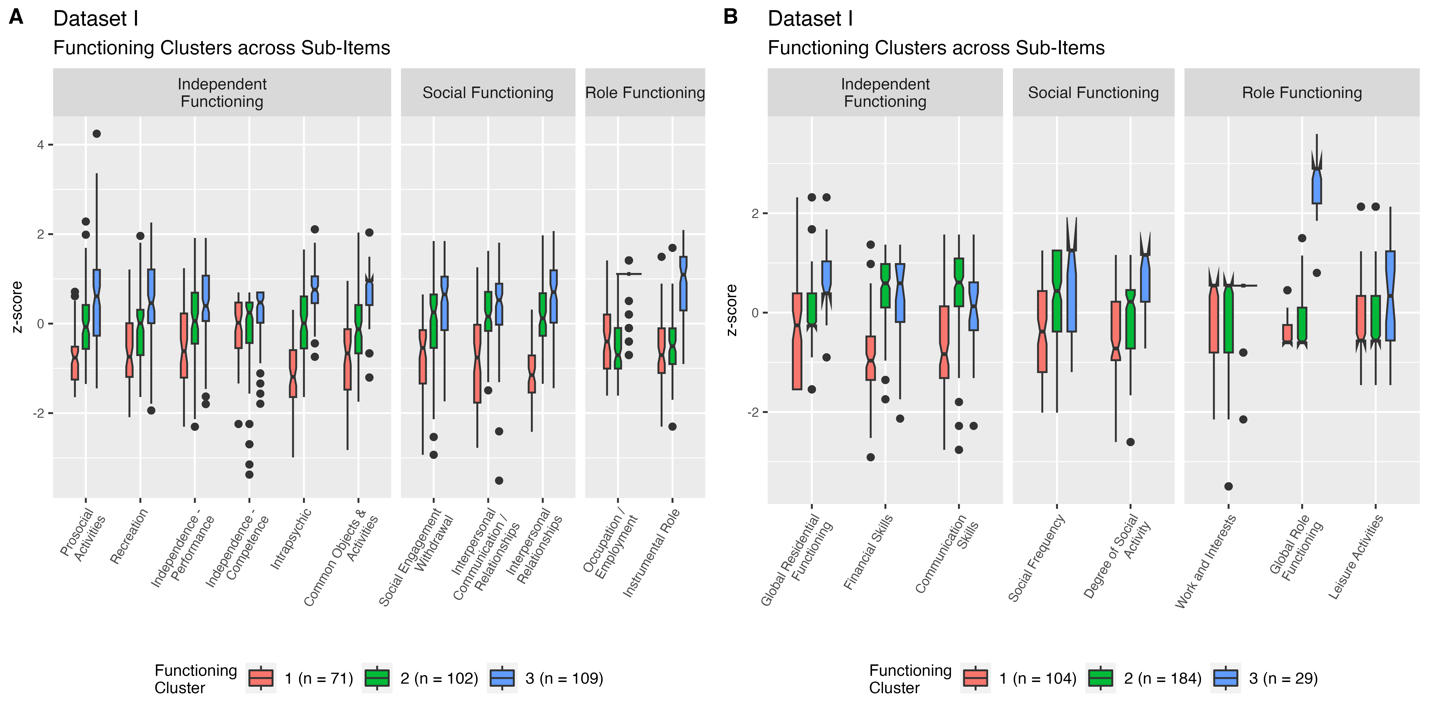


#### Supplemental References

1. Birchwood M, Smith J, Cochrane R, Wetton S, Copestake S. The Social Functioning Scale. The development and validation of a new scale of social adjustment for use in family intervention programmes with schizophrenic patients. Br J Psychiatry. 1990;157:853–9.

2. Heinrichs DW, Hanlon TE, Carpenter WT Jr. The Quality of Life Scale: An Instrument for Rating the Schizophrenic Deficit Syndrome. Schizophrenia Bulletin. 1984 Jan 1;10(3):388–98.

3. Hamilton M. A rating scale for depression. J Neurol Neurosurg Psychiat. 1960;23:56–62.

4. Patterson TL, Mausbach BT. Measurement of Functional Capacity: A New Approach to Understanding Functional Differences and Real-World Behavioral Adaptation in Those with Mental Illness. Annu Rev Clin Psychol. 2010 Mar 1;6(1):139–54.

5. Weissman MM, Olfson M, Gameroff MJ, Feder A, Fuentes M. A Comparison of Three Scales for Assessing Social Functioning in Primary Care. Am J Psychiatry. 2001;

6. Heinrichs DW, Hanlon TE, Carpenter WT Jr. The Quality of Life Scale: An Instrument for Rating the Schizophrenic Deficit Syndrome. Schizophrenia Bulletin. 1984 Jan 1;10(3):388–98.

7. Gonzalez JS, Shreck E, Batchelder A. Hamilton Rating Scale for Depression (HAM-D). In: Gellman MD, Turner JR, editors. Encyclopedia of Behavioral Medicine [Internet]. New York, NY: Springer New York; 2013. p. 887–8. Available from: https://doi.org/10.1007/978-1-4419-1005-9_198

8. Jaeger J, Berns SM, Czobor P. The Multidimensional Scale of Independent Functioning: A New Instrument for Measuring Functional Disability in Psychiatric Populations. Schizophrenia Bulletin. 2003 Jan 1;29(1):153–67.

9. Zweig RA, Turkel E. The Social Adjustment Scale-Self-Report: Psychometric Properties for Older Adults. Psychol Rep. 2007 Dec 1;101(3):920–6.

10. Patterson TL, Goldman S, McKibbin CL, Hughs T, Jeste DV. UCSD Performance-Based Skills Assessment: Development of a New Measure of Everyday Functioning for Severely Mentally Ill Adults. Schizophrenia Bulletin. 2001 Jan 1;27(2):235–45.

11. Davis MH. Measuring individual differences in empathy: Evidence for a multidimensional approach. Journal of Personality and Social Psychology. 1983;44:113–26.

12. Raine A. The SPQ: A Scale for the Assessment of Schizotypal Personality Based on DSM-III-R Criteria. Schizophrenia Bulletin. 1991 Jan 1;17(4):555–64.

13. Overall JE, Gorham DR. The Brief Psychiatric Rating Scale. Psychol Rep. 1962 Jun 1;10(3):799–812.

14. Shafer A. Meta-analysis of the brief psychiatric rating scale factor structure. Psychol Assess. 2005 Sep;17(3):324–35.

15. Andreasen NC. The Scale for the Assessment of Negative Symptoms (SANS): conceptual and theoretical foundations. Br J Psychiatry Suppl. 1989;7:49–58.

16. Nuechterlein KH, Green MF, Kern RS, Baade LE, Barch DM, Cohen JD, et al. The MATRICS Consensus Cognitive Battery, Part 1: Test Selection, Reliability, and Validity. AJP. 2008 Feb 1;165(2):203–13.

17. Wechsler D. Wechsler test of adult reading: WTAR. Psychological Corporation; 2001.

18. Mayer JD, Salovey P, Caruso DR, Sitarenios D. Measuring emotional intelligence with the MSCEIT V2.0. Emotion. 2003;3:97–105.

19. Moore TM, Reise SP, Gur RE, Hakonarson HH, Gur RC. Psychometric properties of the penn computerized neurocognitive battery. Neuropsychology. 2015;29 2:235–46.

20. McDonald S, Flanagan S, Rollins J, Kinch J. TASIT: A New Clinical Tool for Assessing Social Perception After Traumatic Brain Injury. The Journal of Head Trauma Rehabilitation. 2003;18:219–38.

21. Baron-Cohen S, Wheelwright S, Hill J, Raste Y, Plumb I. The “Reading the Mind in the Eyes” Test Revised Version: A Study with Normal Adults, and Adults with Asperger Syndrome or High-functioning Autism. Journal of Child Psychology and Psychiatry. 2001 Feb 1;42(2):241–51.

22. Sergi MJ, Fiske AP, Horan WP, Kern RS, Kee KS, Subotnik KL, et al. Development of a measure of relationship perception in schizophrenia. Psychiatry Research. 2009 Mar 31;166(1):54–62.

23. Dale AM, Fischl B, Sereno MI. Cortical Surface-Based Analysis: I. Segmentation and Surface Reconstruction. NeuroImage. 1999 Feb 1;9(2):179–94.

24. Charrad M, Ghazzali N, Boiteau V, Niknafs A. NbClust: An R Package for Determining the Relevant Number of Clusters in a Data Set. J Stat Soft. 2014 Nov 3;61(6):1–36.

25. Hennig C. Flexible Procedures for Clustering (fps) [Internet]. 2024. Available from: https://cran.r-project.org/web/packages/fpc/index.html

26. Revelle W. psych: Procedures for Psychological, Psychometric, and Personality Research [Internet]. 2022 [cited 2023 Feb 16]. Available from: https://CRAN.R-project.org/package=psych

27. Buuren S van, Groothuis-Oudshoorn K, Vink G, Schouten R, Robitzsch A, Rockenschaub P, et al. mice: Multivariate Imputation by Chained Equations [Internet]. 2022 [cited 2023 Feb 16]. Available from: https://CRAN.R-project.org/package=mice

28. Venables WN, Ripley BD. Modern applied statistics with S [Internet]. 4th ed. New York: Springer; 2002. Available from: https://www.stats.ox.ac.uk/pub/MASS4/

29. Friedman J, Hastie T, Tibshirani R, Narasimhan B, Tay K, Simon N, et al. glmnet: Lasso and Elastic-Net Regularized Generalized Linear Models [Internet]. 2022 [cited 2023 Feb 16]. Available from: https://CRAN.R-project.org/package=glmnet

30. Kassambara A. rstatix: Pipe-Friendly Framework for Basic Statistical Tests [Internet]. 2023 [cited 2023 Feb 16]. Available from: https://CRAN.R-project.org/package=rstatix

31. Holm S. A Simple Sequentially Rejective Multiple Test Procedure. Scandinavian Journal of Statistics. 1979;6(2):65–70.

B
